# Multi-organ landscape of therapy-resistant melanoma

**DOI:** 10.1038/s41591-023-02304-9

**Published:** 2023-04-27

**Authors:** Sixue Liu, Prashanthi Dharanipragada, Shirley H. Lomeli, Yan Wang, Xiao Zhang, Zhentao Yang, Raymond J. Lim, Camelia Dumitras, Philip O. Scumpia, Steve M. Dubinett, Gatien Moriceau, Douglas B. Johnson, Stergios J. Moschos, Roger S. Lo

**Affiliations:** 1 Division of Dermatology, Department of Medicine, David Geffen School of Medicine, University of California, Los Angeles, Los Angeles, CA USA; 2 Department of Molecular and Medical Pharmacology, David Geffen School of Medicine, University of California, Los Angeles, Los Angeles, CA USA; 3 Division of Pulmonary, Critical Care and Sleep Medicine, Department of Medicine, David Geffen School of Medicine, University of California, Los Angeles, Los Angeles, CA USA; 4 Jonsson Comprehensive Cancer Center, David Geffen School of Medicine, University of California, Los Angeles, Los Angeles, CA USA; 5 Department of Dermatology, Veterans Administration Greater Los Angeles Healthcare System, Los Angeles, CA USA; 6 Division of Hematology/Oncology, Department of Medicine, Vanderbilt University Medical Center, Nashville, TN USA; 7 Vanderbilt Ingram Cancer Center, Vanderbilt University Medical Center, Nashville, TN USA; 8 Division of Medical Oncology, Department of Medicine, The University of North Carolina at Chapel Hill, Chapel Hill, NC USA; 9 Lineberger Comprehensive Cancer Center, The University of North Carolina at Chapel Hill, Chapel Hill, NC USA

**Keywords:** Melanoma, Translational research, Cancer genomics, Oncogenesis, Metastasis

## Abstract

Metastasis and failure of present-day therapies represent the most common causes of mortality in patients with cutaneous melanoma. To identify the underlying genetic and transcriptomic landscapes, in this study we analyzed multi-organ metastases and tumor-adjacent tissues from 11 rapid autopsies after treatment with MAPK inhibitor (MAPKi) and/or immune checkpoint blockade (ICB) and death due to acquired resistance. Either treatment elicits shared genetic alterations that suggest immune-evasive, cross-therapy resistance mechanisms. Large, non-clustered deletions, inversions and inter-chromosomal translocations dominate rearrangements. Analyzing data from separate melanoma cohorts including 345 therapy-naive patients and 35 patients with patient-matched pre-treatment and post-acquired resistance tumor samples, we performed cross-cohort analyses to identify MAPKi and ICB as respective contributors to gene amplifications and deletions enriched in autopsy versus therapy-naive tumors. In the autopsy cohort, private/late mutations and structural variants display shifted mutational and rearrangement signatures, with MAPKi specifically selecting for signatures of defective homologous-recombination, mismatch and base-excision repair. Transcriptomic signatures and crosstalks with tumor-adjacent macroenvironments nominated organ-specific adaptive pathways. An immune-desert, CD8^+^-macrophage-biased archetype, T-cell exhaustion and type-2 immunity characterized the immune contexture. This multi-organ analysis of therapy-resistant melanoma presents preliminary insights with potential to improve therapeutic strategies.

## Abstract (web summary)

Genomic analyses in a rapid autopsy cohort study of patients with melanoma identify the genetic and transcriptomic landscape of melanoma with acquired resistance to MAPK inhibitor and immune checkpoint blockade therapies, providing insights for the potential improvement of therapeutic strategies.

## Main

Cutaneous melanoma (CM) exhibits a UV-related high mutational burden^1,2^. Mutually exclusive *BRAF* and *NRAS* mutations drive MAPK addiction in ~70% of metastatic CM^3^. CM genomes also harbor a high burden of structural variants (SVs)^4^ and chromothripsis^5^. Current knowledge of this mutational landscape is derived from tumors naive to highly active treatments developed recently and inclusive of earlier-stage disease. How MAPK inhibitor (MAPKi)/immune checkpoint blockade (ICB) therapies alter the mutational landscape and, thereby, cause death remains largely unknown.

Metastases cause most cancer-related deaths^6^, and CM is among the most metastatic (>60% of autopsies with brain metastases)^7,8^. Despite experimental metastasis studies, the difficulty of accessing patient-derived metastatic tissues has limited understanding of clinical metastatic and organ-specific evolution. Although the concept of an organ-specific pre-metastatic niche has been demonstrated experimentally^9^, little is known clinically regarding co-adaptations between metastases and their site-specific macroenvironments.

Although MAPKi/ICB have become standard-of-care therapies for patients with metastatic CM in developed countries, clinical relapse occurs commonly, with multi-therapy resistance being highly lethal. Acquired MAPKi resistance in CM has been evaluated at an omics scale in a few cohorts, but knowledge of clinically acquired ICB resistance is limited^10–18^. Metastases to accessible anatomic sites overrepresent current datasets on acquired resistance. Monitoring (for example, liquid biopsy) and therapeutic strategies to counter resistance require insights into multi-organ mechanisms and heterogeneity. Whole-exome sequencing (WES) and whole-genome sequencing (WGS) can characterize patterns termed ‘mutational signatures’ that reflect imprints of DNA mutagenic processes and defective DNA damage repair processes^19,20^. It is unknown whether rare signatures of a particular malignancy, such as UV-related CM, are common with respect to late mutations, potentially due to the influence of a particular therapy. Such signatures that emerge later during tumor evolution might represent targetable pathway defects or synthetic lethalities.

Hence, we assembled a rapid autopsy melanoma (RAM) cohort from patients with *BRAF*^MUT^ or *NRAS*^MUT^ CM who were treated with and responded initially to MAPKi/ICB therapies but later died because of disease progression. This cohort includes 71 distinct metastatic tumors, 41 tumor-adjacent ‘normal’ (AN) tissues representing organ-specific tumor macroenvironments and 38 tumor-non-adjacent normal (NAN) tissues. We generated and analyzed WES from tumors and patient-matched NANs as well as WGS from a subset. To dissect the contribution of distinct therapies, we comparatively analyzed WES data from longitudinal pre- and post- tumors from patients with CM who had progressed on either MAPKi-only or ICB-only therapy. Moreover, we developed organ-specific metastatic signatures based on tumorcell-enriched transcriptomes and analyzed ligand–receptor signaling between tumor and AN tissues. Finally, we deconvolved tumor, AN and NAN transcriptomes to decipher organ-specific immune contextures.

## Results

### RAM cohort, omic data and comparative cohorts

**Extended Data Fig. 1:**
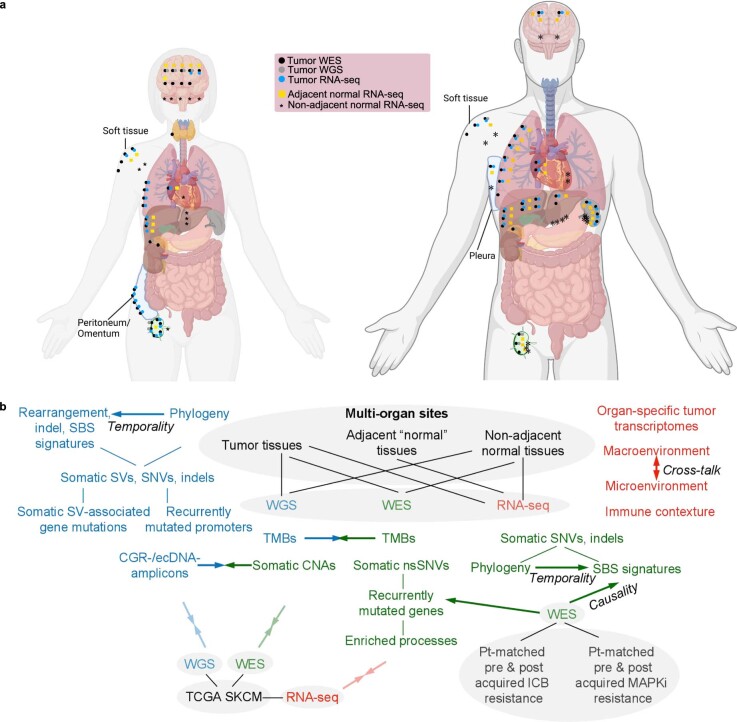
Overview of the tissues sampled at rapid autopsies and the study design. (**a**) Numbers and types of tissues, sequencing strategies, and metastatic sites are shown for 11 rapid melanoma autopsy (RAM) cases consisting of five deceased female patients and six deceased male patients with *BRAF*^MUT^ (n = 7) or *NRAS*^MUT^ (n = 4) cutaneous melanoma. Not represented are RNA-seq derived from triplicates and WES derived from non-adjacent normal tissues. (**b**) Inclusion of tissue sets and datasets (grey background) and flow chart of analyses performed. RAM is the core tissue or data set, supplemented by comparisons (transparent, adjoined arrows pointing in opposite directions) with two tissue/data sets. RAM cohort (tumor, n = 22 for WGS, n = 74 for WES, n = 93 for RNA-seq; adjacent ‘normal’, n = 68 for RNA-seq; non-adjacent normal, n = 67 for RNA-seq, n = 10 for WES). Pre-and-post cohort consists of patient-matched cutaneous melanoma tumors biopsied pretreatment and post acquired resistance (ICB, n = 7 pairs; MAPKi, n = 59 pairs; both subgroups with patient-matched normal tissues for all patients; n = 102 tumor WES datasets). TCGA–SKCM cohort, only *BRAF*^MUT^ (n = 233) or *NRAS*^MUT^ (n = 125) tumors included (13 tumors with both *BRAF* and *NRAS* mutations; total n = 345 tumors). Bolded arrows, analyses compared to derive insights into temporality or causality.

From 11 RAM cases, we collected (2012–2019) tumor, AN (≤1 cm from tumor border) and NAN (>1 cm) tissues from deceased patients (five females and six males, all of European ancestry; seven *BRAF*^MUT^ and four *NRAS*^MUT^; five of 11 with radiation-treated brain metastasis) (Supplementary Table 1 and Extended Data Fig. 1a). All patients before autopsies progressed on the last therapy with nearly all tumors acquiring resistance, went to hospice and died. Four patients had been treated with only MAPKi; four with only ICB; and three with MAPKi and ICB (in sequence) (Supplementary Table 1). We generated WES (Supplementary Table 2) from 74 tumors (three multi-regional samples) (brain, thyroid, heart or cardio, lung, liver/gallbladder, spleen, adrenal, lymph node (LN), soft tissues (ST) and pleural membrane or the omentum/peritoneum) and 10 patient-matched normals (Extended Data Fig. 1a). We also generated WGS (Supplementary Table 3) from 22 tumors (brain, lung, liver, adrenal, LN and ST) (Extended Data Fig. 1a). Finally, we generated RNA sequencing (RNA-seq) from 93 tumors, 68 ANs and 67 NANs (inclusive of multi-regional samples) from all sites except thyroid (Extended Data Fig. 1a and Supplementary Table 4). We comparatively analyzed (Extended Data Fig. 1b) CM data from (1) TCGA–SKCM^2^, (2) patient-matched pre and post tumors with MAPKi-only or ICB-only treatments (Supplementary Table 5) and (3) matched vehicle-treated/MAPKi-sensitive and acquired MAPKi-resistant patient-derived xenografts (PDXs) (Supplementary Table 6).

### Somatic exomic alterations

**Fig. 1:**
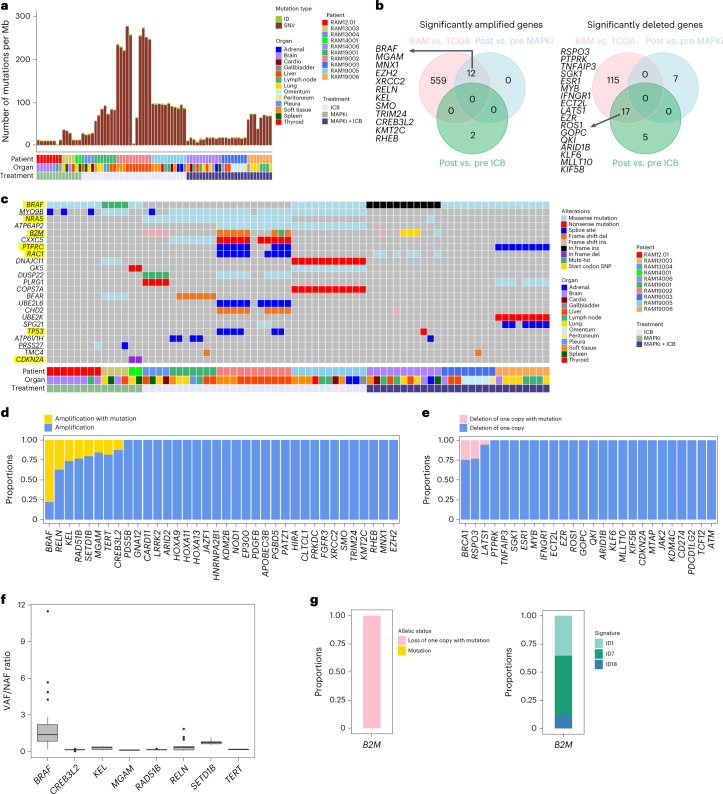
Whole-exomic landscape of therapy-resistant cutaneous melanoma. **a**, Tumor somatic mutational burdens based on synonymous and non-synonymous IDs and SNVs across RAM cases (patients), organ sites and treatment histories. **b**, Venn diagrams showing overlaps of significantly amplified (left) or deleted (right) genes based on frequency-enriched CNAs observed in three comparative cohorts: (1) RAM tumors versus TCGA–SKCM tumors (*BRAF* mutant and *NRAS* mutant only); (2) MAPKi-only post versus pre tumors; and (3) ICB-only post versus pre tumors. **c**, Highly expressed SMGs in the RAM tumor cohort annotated by mutational types, cases, organ sites and treatment histories. Yellow highlights, SMGs identified by prior published studies (Extended Data Fig. 2g,h). Underlines, SMGs significantly (two-sided Fisher’s exact test, FDR-adjusted *P* < 0.05) enriched in the RAM tumor cohort (versus the TCGA–SKCM tumor cohort, *BRAF* mutant and *NRAS* mutant only). *MYO9B*, *P* = 7.97 × 10^−5^ and adjusted *P* = 0.004144; *B2M*, *P* = 1.75 × 10^−3^ and adjusted *P* = 0.044013; *PRSS27*, *P* = 2.12 × 10^−3^ and adjusted *P* = 0.044013. **d**,**e**, Copy number and mutational (missense, nonsense, splice-site, frame-shift IDs) status of overlapping amplified (**d**) or deleted (**e**) genes (**b** and Extended Data Fig. 2c). **f**, Ratios of variant versus normal allele frequencies in eight overlapping amplified genes (*BRAF*, *n* = 39; *CREB3L2*, *n* = 7; *KEL*, *n* = 15; *MGAM*, *n* = 9; *RAD51B*, *n* = 5; *RELN*, *n* = 26; *SETD1B*, *n* = 6; *TERT*, *n* = 5 tumors). Central line of each box, median; top and bottom edges of each box, first and third quartiles; whiskers extend 1.5× the interquartile range beyond box edges. **g**, Copy number and mutational status of the overlapping SMG *B2M* (Extended Data Fig. 2e) in the RAM cohort (left); ID signatures identified among *B2M*-mutated RAM tumors (right). del, deletion; ins, insertion; SNP, single-nucleotide polymorphism. Source data

**Extended Data Fig. 2:**
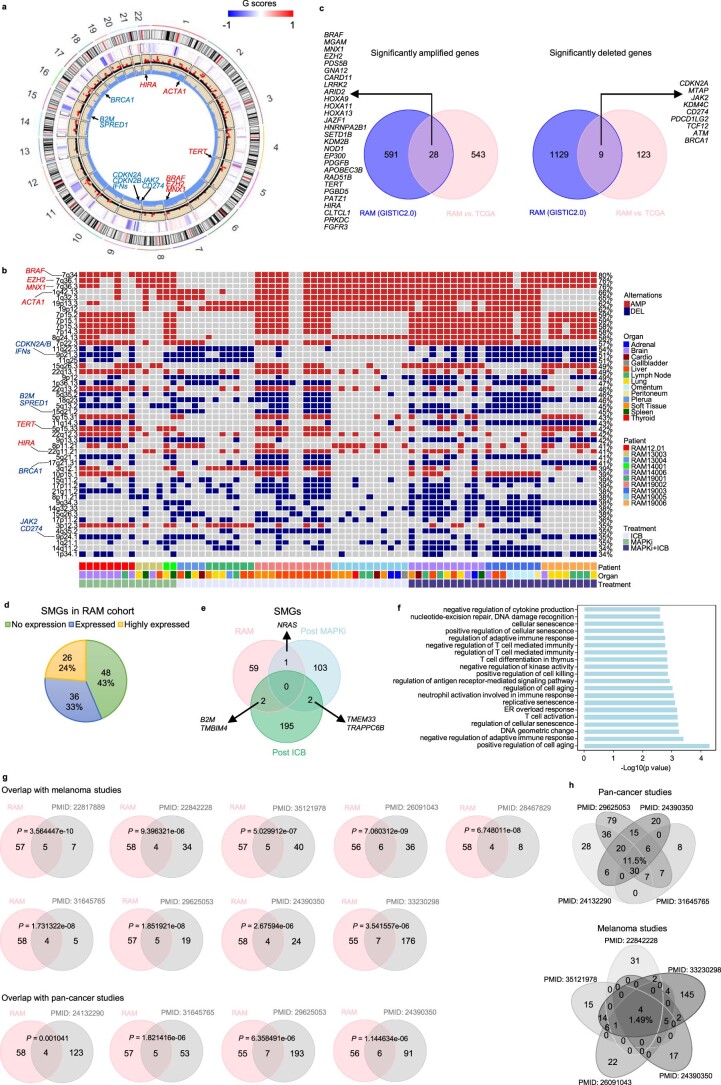
Significant copy-number alterations and mutated genes enriched in the RAM tumor cohort. (**a**) Somatic copy-number alterations of the RAM cohort (n = 74 tumors) (left). Outermost layer, chromosomal regions; second layer, heatmap of G scores (GISTIC 2.0); third layer, *P* values of amplified and deleted regions (red dots indicate significance); innermost layer, frequencies of amplified and deleted regions; arrows, locations of functionally important genes displaying significant amplification (red) or deletion (blue). (**b**) Top 50 significantly altered exomic regions by GISTIC analysis, listed in decreasing order of recurrence and annotated with associated genes, across RAM cases/patients, organ sites, and treatment histories. AMP, amplification; DEL, deletion. (**c**) Venn diagrams showing overlaps between (i) significantly amplified (left) or deleted (right) genes based on GISTIC2.0 analysis of the RAM tumors and (ii) frequency-enriched, copy number alterations observed in RAM tumors versus TCGA–SKCM tumors (*BRAF*- and *NRAS*-mutant only). (**d**) The numbers and proportions of significantly mutated genes (SMGs) with no expression (mean log_2_CPM < 0), expresssion (0 ≤ mean log_2_CPM < 4), or high expression (mean log_2_CPM > 4) among RAM tumors. (**e**) Venn diagram of overlapping SMGs identified in three tumor cohorts: (i) RAM, (ii) clinical post-MAPKi-only (pre-MAPKi SMGs subtracted) and, (iii) clinical post-ICB-only (pre-ICB SMGs subtracted). (**f**) Top 20 significantly enriched biological processes of SMGs with expression (as defined in d). One-sided Fisher’s exact test without multiple comparisons. (**g**) Venn diagrams showing overlapping numbers of SMGs identified in the RAM tumor cohort versus SMGs identified in published (PMIDs shown) large-scale melanoma and pan-cancer cohorts. *P* values by one-sided hypergeometric test. (**h**) Venn diagrams showing overlapping numbers of SMGs identified in published pan-cancer (top) or melanoma (bottom) cohorts. Source data

At a median coverage of 217×, the mean number of somatic mutations/tumor and tumor mutational burden (TMB) was 4,401 (range, 167–17,872) and 68.8 mutations per megabase (Mb) (range, 2.6−279.3) (Fig. 1a and Supplementary Table 2). For non-synonymous somatic mutations, a mean of 675 mutations per tumor (range, 24–2,669) corresponded to a TMB of 22.49 mutations per Mb (range, 0.8–88.97) (Supplementary Table 2). These TMBs are higher than or similar to previous estimates (Supplementary Table 7). We detected by Genomic Identification of Significant Targets In Cancer (GISTIC2.0) 48 significantly amplified and 75 significantly deleted regions (Extended Data Fig. 2a and Supplementary Table 8). Amplified regions harbored *BRAF* (80% of tumors), *ACTA1* (66%) and *TERT* (42%); deleted regions harbored *IFN* cluster genes and *CDKN2A/B* (51%), *B2M* and *SPRED1* (45%), *BRCA1* (41%) and *JAK2* and *CD274* (35%) (Extended Data Fig. 2b). Tumors from six of six MAPKi-only and MAPKi+ICB cases harbored *BRAF* amplification, whereas tumors from two of four ICB-only cases harbored *BRAF* amplification. Copy number loss of *JAK2*, previously associated with acquired ICB resistance^21,22^, was observed in MAPKi-only cases. The findings of mechanisms of acquired MAPKi resistance in tumors with acquired ICB resistance and vice versa suggest cross-resistant mechanisms that converge on immune evasion.

To match RAM tumors, we selected TCGA–SKCM tumors driven by *BRAF* or *NRAS* mutations. By comparing RAM versus TCGA–SKCM copy number alteration (CNA) frequencies with Fisher’s exact test, we detected 571 significantly amplified and 132 significantly deleted genes in the RAM cohort (Supplementary Table 9). We observed significant overlaps of amplified (but not deleted) genes between findings from GISTIC and RAM-enriched (versus TCGA–SKCM) CNAs (*P* = 0.0482198, hypergeometric test) (Extended Data Fig. 2c). We tested the hypothesis that a subset of RAM-enriched (versus TCGA–SKCM) CNAs is due to acquired resistance to MAPKi/ICB therapy. We identified significant CNAs in post-treatment (versus pre-treatment) melanoma from patients with either MAPKi-only or ICB-only treatment (between pre and post biopsies) histories. Notably, gene amplifications overlapped significantly between the RAM (versus TCGA–SKCM) and the MAPKi-only post (versus pre) frequency-enriched CNAs (*P* = 1.205907 × 10^−18^, hypergeometric test) (Fig. 1b). In contrast, gene deletions overlapped significantly between the RAM (versus TCGA–SKCM) and the ICB-only post (versus pre) frequency-enriched CNAs (*P* = 6.52371 × 10^−33^, hypergeometric test) (Fig. 1b). Thus, acquired resistance to ICB versus MAPKi distinctly contributes to the RAM CNA landscape.

We also nominated significantly mutated genes (SMGs) (Fig. 1c and Supplementary Table 10). To circumvent a limited cohort size, we inflated type I error by not performing multiple testing but reduced false positives by enforcing an expression cutoff. MutSig2CV (at a raw *P* < 0.05 cutoff) called 110 SMGs. Among these, we nominated 62 SMGs (Extended Data Fig. 2d) with RNA (Supplementary Table 10) or high RNA (Fig. 1c and Supplementary Table 10) expression, using cohort-matched transcriptomes. Among high-expression SMGs, we identified resistance driver mutations in *BRAF* and *NRAS* and predicted loss-of-function *B2M* mutations (Fig. 1c). Consistent with resistance causation, *B2M* was significantly mutated in ICB-only post (but not pre) tumors (Extended Data Fig. 2e). Moreover, the 62 nominated SMGs enriched for immune, cell death and senescence regulation (Extended Data Fig. 2f). We cross-referenced the 62 RAM-nominated SMGs to SMGs reported by large-scale, melanoma-specific or pan-cancer studies and observed highly significant overlaps (Extended Data Fig. 2g). As context, among these publications, the consensus SMGs comprised small fractions (1.49% and 11.5% among melanoma and pan-cancer cohorts, respectively) (Extended Data Fig. 2h). Seven high-expression RAM SMGs (*BRAF*, *NRAS*, *B2M*, *PTPRC*, *RAC1*, *TP53* and *CDKN2A*) were previously reported as SMGs (Fig. 1c).

Among genes affected by overlapping CNAs (Fig. 1b and Extended Data Fig. 2c), eight amplified genes harbored mutations in at least one copy (Fig. 1d,e). *BRAF* displayed a mean ratio of variant-to-normal allelic frequency of 1.87 (Fig. 1f), indicating selective amplification of the mutant allele. Among affected tumors, deleted genes predominantly showed single-copy loss (Fig. 1e). Several genes (*BRCA1*, *RSPO3* and *LATS1*) were affected by both deletions and mutations. Among *B2M-*mutated tumors, 100% harbored bi-allelic loss-of-function alterations (deletion of one and mutation of another copy) (Fig. 1g). Because *B2M* mutations consisted of small insertions and deletions (IDs), we found enrichment of ID1 and ID7 signatures among *B2M*-mutated tumors (Fig. 1g), suggesting defective DNA mismatch repair (MMR) as a cause.

### Phylogeny and heterogeneity

**Fig. 2:**
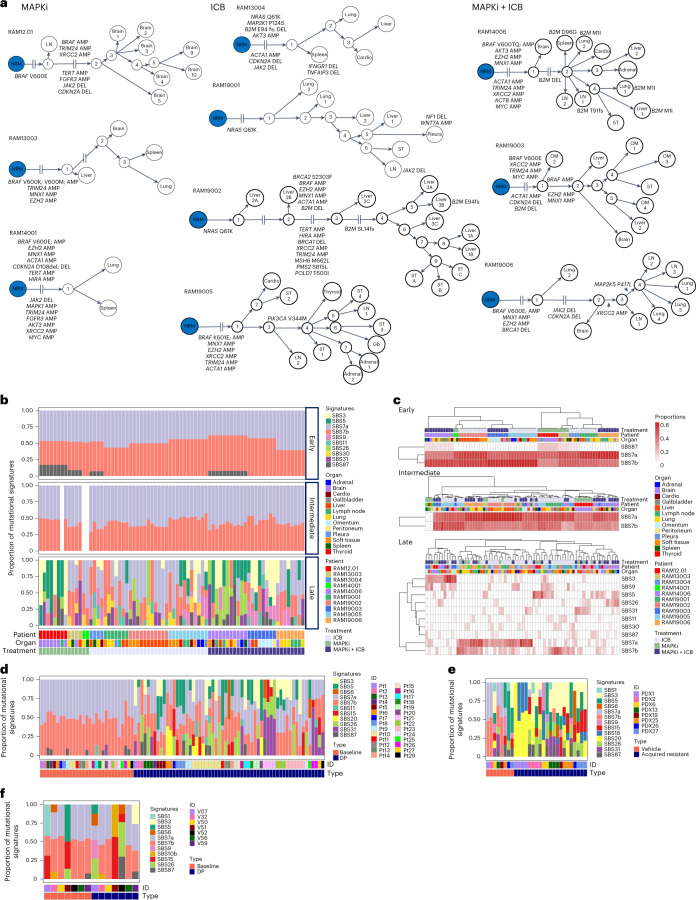
Multi-organ temporal mutational patterns of RAM tumors. **a**, Phylogenetic relationship of multi-organ metastases in 10 RAM cases organized by treatment histories. Each tumorʼs somatic mutations (SNVs and IDs) were used to construct a maximally parsimonious phylogenetic tree. Some branches, as indicated, are not shown to scale owing to extensive lengths. Each evolutionary trajectory is annotated by selected cancer genes and their mutations. AMP, copy number amplification; DEL, copy number deletion; Gb, gallbladder; NRM (blue node), normal tissue; OM, omentum. **b**, Spectra of mutational signatures among early, intermediate and late mutations, based respectively on shared, semi-private and private SBSs in **a** across RAM cases, organ sites and treatment histories. With WES of two RAM14001 tumors available, we identified only early and late mutations for signature detection. **c**, Unsupervised clustering of treatment histories, RAM cases and organ sites based on the proportions or compositions of SBS mutational signatures present in early, intermediate and late mutations in **b**. **d**, Analysis of SBS mutational signatures in a clinical cohort of patient-matched MAPKi-sensitive (referred to as baseline) and MAPKi-acquired resistant (referred to as disease progression (DP)) cutaneous *BRAF*^MUT^ melanoma tumors (*n* = 88 tumors). MAPKi-sensitive tumors represent ICB-naive, pre-MAPKi-treatment clinical tumors (*n* = 28 patients). **e**, As in **d**, except all samples are PDX tumors from sex-matched NSG mice and consisting of patient-matched (*n* = 8 models), vehicle-treated and MAPKi-sensitive tumors (*n* = 8) and acquired MAPKi-resistant *BRAF*-mutant or *NRAS*-mutant tumors (*n* = 21). **f**, As in **d**, except all samples are clinical tumors (*n* = 14) consisting of patient-matched MAPKi-naive (except one), pre-ICB baseline tumors and acquired ICB-resistant tumors (*n* = 7 patients). Source data

**Extended Data Fig. 3:**
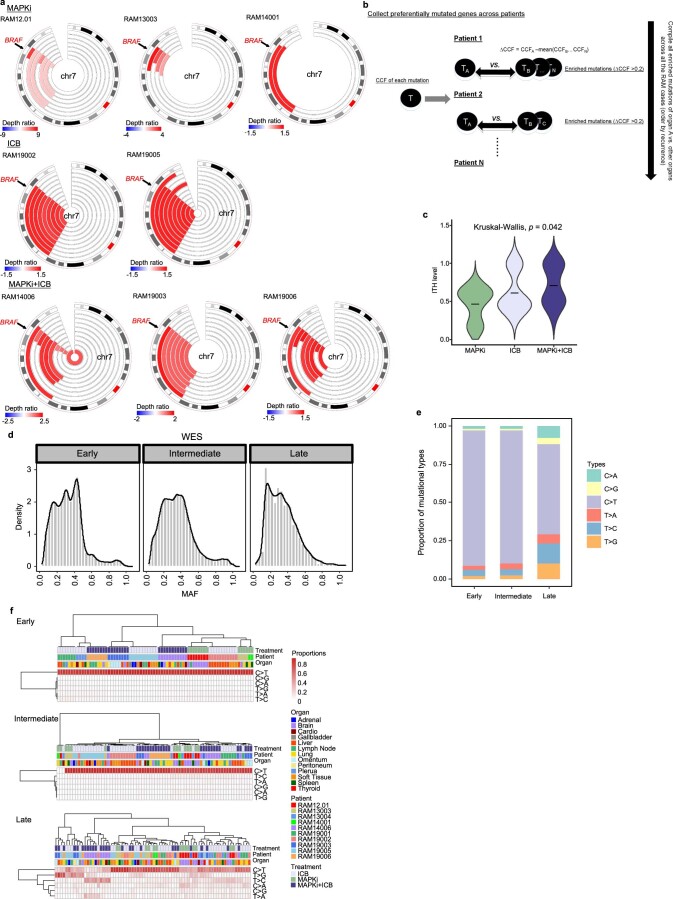
Cancer cell fractions and mutational spectra analysis. (**a**) Circos representation of copy number gains harboring *BRAF* in multiple metastases from eight RAM cases. For each Circos plot (RAM case), the outermost layer represents chromosome 7 regions, and the inner heatmaps indicate copy number status (spanning region and depth ratio) of tumors with *BRAF* amplification. Arrow indicates the location of *BRAF*. (**b**) Schema of sub-clonal analysis of multi-organ metastases to identify organ-specific enrichment of mutations. Each mutation’s cancer cell fraction (CCF) is estimated using PyClone-VI. The ∆CCF of each mutation was calculated between tumors of organ A versus tumors of other organs in each RAM case. Enriched mutations in organ A are defined as those with a ∆CCF difference > 0.2. The recurrence of genes harboring organ A-enriched mutations is computed by combining the enriched mutations of tumors from organ A across all applicable RAM cases. (**c**) Intratumoral heterogeneity (**ITH**) levels (fraction of sub-clonal mutations per tumor) of RAM tumors by treatment histories. *P* values, Kruskal–Wallis test. ICB, n = 33; MAPKi, n = 14; ICB + MAPKi, n = 27. (**d**) Distribution of mutant allele frequencies (MAF) of early, intermediate, and late mutations. (**e**) Mutational spectra of early, intermediate, and late mutations. (**f**) Unsupervised clustering of mutational spectra among early, intermediate, and late mutations across treatment histories, RAM cases/patients, and organ sites. Source data

RAM tumors evolved via both linear and branched divergence without any organ-specific pattern (Fig. 2a). Regardless of treatment history, prominent truncal amplifications involved *BRAF*, *MNX1*, *EZH2*, *XRCC2* and *ACTA*, and truncal deletions involved *JAK2* and *CDKN2A* (Fig. 2a and Supplementary Table 1). In contrast, *B2M* somatic mutations and deletions were exclusive to ICB-only and MAPKi+ICB cases and occurred as truncal, semi-truncal or private events (Fig. 2a). Loss-of-function mutations affecting *JAK2*, *CDKN2A* (and other 9p21.3 genes—for example, IFN-induced genes; Extended Data Fig. 2a) and *B2M* indicated immune evasion as mechanisms of acquired resistance^3,10,23^. In RAM19002, non-synonymous mutations of MMR genes (*MSH6*, *PMS2* and *POLD1*) preceded *B2M* IDs (Fig. 2a). We also observed convergent evolution due to MAPKi/ICB therapies (for example, in RAM14006, distinct, second-hit non-synonymous *B2M* mutations (Fig. 2a); in multiple cases, distinct *BRAF* amplicon boundaries (Extended Data Fig. 3a)). By analyzing tumor cell fractions of mutated genes, we failed to detect organ-specific enrichment for specific mutated genes (Extended Data Fig. 3b). Finally, intratumoral heterogeneity (ITH), estimated by the proportion of subclonal mutations, was significantly lower after MAPKi-only treatment (Extended Data Fig. 3c).

### Altered mutational spectra and signatures

We previously identified altered mutational spectra associated with somatic mutations unique to acquired MAPKi resistance^14^. Here, we divided somatic mutations into early, intermediate and late mutations and analyzed mutant allelic frequencies (Extended Data Fig. 3d), mutational spectra (Extended Data Fig. 3e) and single-base substitution (SBS) signatures based on COSMIC version 3.3 (Fig. 2b). The mean mutant allelic frequencies of early, intermediate and late somatic single-nucleotide variants (SNVs) were, respectively, 0.39, 0.36 and 0.27. Early and intermediate mutational spectra displayed a case-specific but no site-specific or treatment-specific pattern. However, late mutational spectra clustered independently of case, site or treatment history (Extended Data Fig. 3f), enriching for C>A, T>C and T>G (Extended Data Fig. 3e). Among 10 SBS signatures, UV signatures (SBS7a and SBS7b) dominated early and intermediate somatic mutations (Fig. 2b) in a case-specific pattern (Fig. 2c). Notably, non-UV-related signatures dominated late mutations (Fig. 2b) with extensive intra-patient and inter-patient heterogeneity but treatment-elicited convergence (for example, signatures of defective homologous recombination repair (HHR) and MMR clustering with MAPKi) (Fig. 2c). Notably, SBS3 (defective HRR) was detected among late mutations in nine of 10 patients (Fig. 2b). We also detected SBS5 (clock-like), SBS9 (polymerase eta somatic hypermutation), SBS26 (defective MMR), SBS30 (defective DNA base excision repair (BER) due to *NTHL1* mutations), SBS31 (platinum treatment) and SBS87 (thiopurine treatment) among late mutations in most patients. SBS11 (temozolomide) was detected among late somatic mutations in association with ICB treatment (Fig. 2b).

We sought to validate an association between non-UV-related signatures and MAPKi/ICB therapies. We assembled two MAPKi validation cohorts of patient-matched melanoma with WES data: (1) a clinical *BRAF*^MUT^ cohort (88 tumors from 28 patients) consisting of pre-treatment and acquired-resistant tumors (along with normal genomic DNA (gDNA)) (Supplementary Table 5) and (2) a PDX *BRAF*^MUT^ or *NRAS*^MUT^ cohort (29 tumors from eight models) consisting of model-matched vehicle-treated and acquired-resistant tumors derived in patient sex-matched NSG mice (information on gender of source patients in Supplementary Table 6) (along with normal gDNAs). All pre-treatment melanomas in both MAPKi cohorts were ICB naive and MAPKi sensitive (Supplementary Tables 5 and 6). We extracted somatic mutations unique to acquired MAPKi resistance and identified the frequencies of SBS signatures, including 12 non-UV signatures of defective HRR, MMR and BER (Fig. 2d,e). In contrast, UV signatures dominated the SNVs in pre/sensitive tumors. We also assembled WES data from patient-matched pre/sensitive and post/acquired ICB-resistant melanoma (14 tumors from seven patients) and normal gDNAs. All pre/sensitive melanoma in this ICB validation cohort, except one (V52), were MAPKi naive (Supplementary Table 5). After extracting SBS signatures from somatic SNVs in pre-treatment tumors and unique to acquired resistance, we detected enrichment of UV signatures in four of seven acquired ICB-resistant tumors (Fig. 2f). Thus, MAPKi (versus ICB) therapy selects for non-UV-related mutational signatures.

### Somatic whole-genomic alterations

**Fig. 3:**
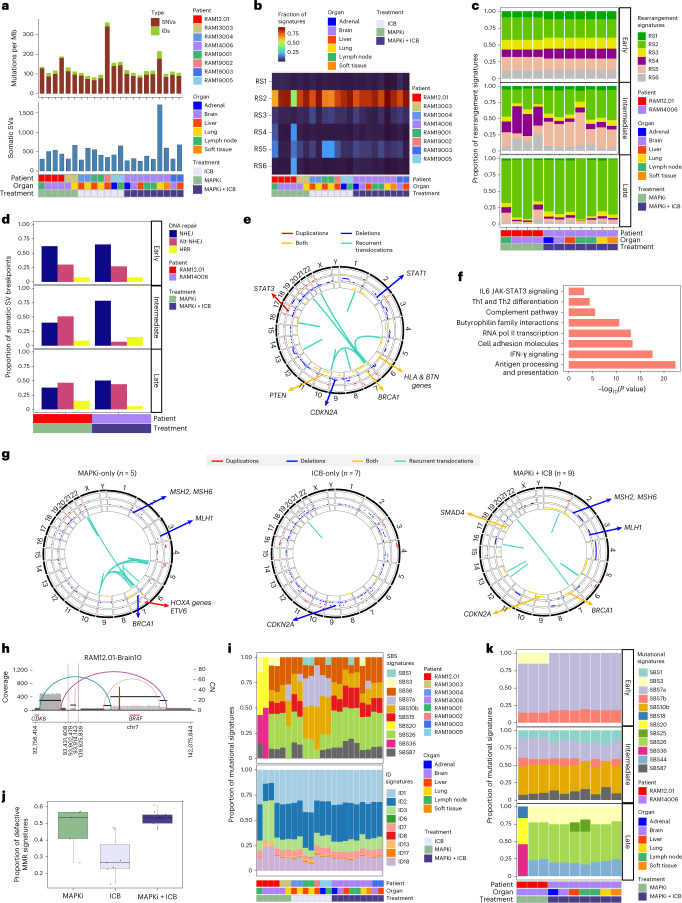
Whole-genomic landscape of therapy-resistant cutaneous melanoma. **a**, TMBs based on somatic IDs, SNVs and SVs across 22 tumors (eight cases), organs and treatment histories. **b**,**c**, Spectra of RSs among all SVs (**b**) or early, intermediate and late SVs (**c**). Tumors analyzed in **c** derived from phylogenetic analysis of two RAM cases with multiple tumors. **d**, Distribution of DNA DSB repair processes (NHEJ, alternative NHEJ and HRR) inferred by breakpoint junctional sequence analysis of early, intermediate and late SVs of RAM cases in **c**. Homologous sequence lengths at breakpoints of 0–1 bp, 2–6 bp or >6 bp infer NHEJ, alternative NHEJ and HRR, respectively. **e**, SVs across genomic locations and affected genes. Outer layer, chromosome locations; second layer, large (>1 Mb) duplications (red); third layer, large deletions (blue); inner layer, a combination of both duplications and deletions (yellow); frequencies of occurrence increase from inner to outer circles. Inside the circles, recurrent (≥4 of 21 resistant RAM tumors) intra-chromosomal or inter-chromosomal translocations. **f**, Pathways enriched in genes overlapping somatic SVs detected in ≥50% (11/21) of RAM tumors (one-sided Fisher’s exact test, adjusted by FDR). **g**, As in **e**, except sub-analyses based on treatment histories and recurrent translocation in ≥3 tumors in each treatment history category. **h**, SV plot indicating extrachromosomal DNA amplicon harboring *BRAF* in a brain metastasis. Horizontal black and red lines indicate, respectively, genomic segments with similar copy numbers and genes. Each line/arc representing discordant reads is colored based on differences from expected distance or orientation. **i**, Spectra of SBS and ID signatures based on WGS across 22 tumors (eight cases), organs and treatment histories. **j**, Proportions of SBS mutational signatures associated with defective MMR, per treatment history (*n* = 22 tumors). Central line of each box, median; top and bottom edges of each box, first and third quartiles; whiskers extend 1.5× the interquartile range beyond box edges. **k**, As in **i**, except for early, intermediate and late SBS mutations in two RAM cases with phylogenetic data. Source data

**Extended Data Fig. 4:**
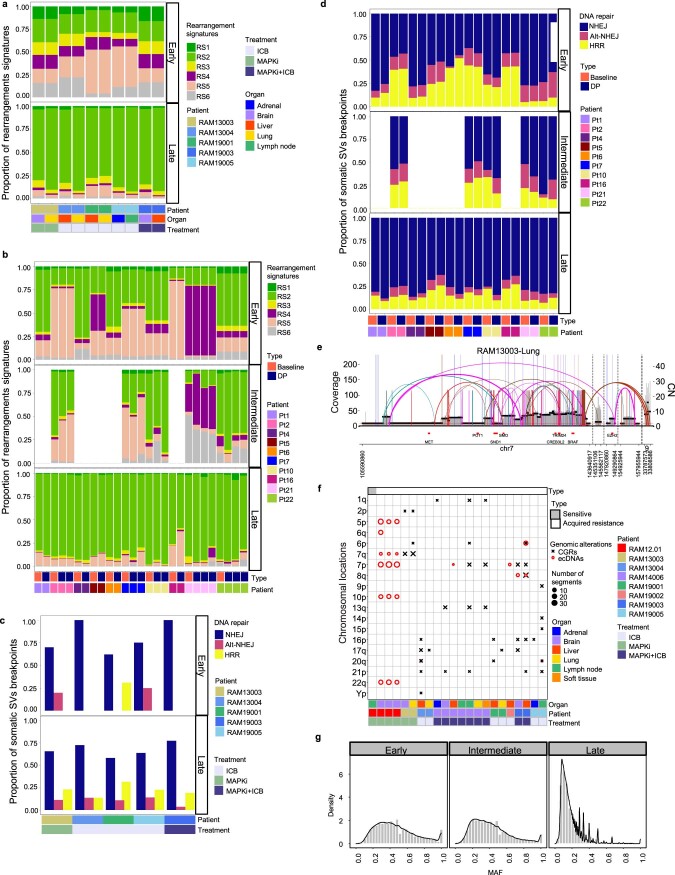
Structural variations, complex genomic rearrangements (CGRs) and extrachromosomal DNAs (ecDNAs) amplicons, and mutations. (**a**) Spectra of rearrangement signatures (RSs) among early or late structural variations (SVs) in RAM cases (n = 5 cases; n = 5 sites). (**b**) Spectra of RSs among early, intermediate, or late SVs in a clinical MAPKi-treated, patient-matched pre-and-post melanoma cohort (n = 10 patients; n = 10 baseline tumors; n = 17 disease progression or DP tumors). (**c**) Distribution of double-stranded DNA break (DSB) repair processes (non-homologous end-joining or NHEJ, alternative NHEJ or alt-NHEJ, homologous recombination repair or HRR) inferred by breakpoint-junctional sequence analysis of early or late SVs of RAM cases in (a). (**d**) Distribution of DSB repair processes inferred by breakpoint-junctional sequence analysis of early, intermediate, or late SVs of the cohort in (b). For cases with multiple DPs, average values are presented. (**e**) SV plot indicating CGR harboring *BRAF* in a lung metastasis. Horizontal black and red lines indicate respectively genomic segments with similar copy numbers and genes. Short vertical lines and arcs indicate discordant read pairs linking two amplicons via a structural variant junction. Long vertical lines indicate break ends that map from amplicon into low-complexity regions which cannot be traced further. Each line/arc representing discordant reads is colored based on differences from expected distance or orientation. (**f**) Chromosomal distribution of ecDNAs and CGRs in RAMs (n = 22; one remained sensitive to treatment; 21 with acquired MAPKi-resistance) across organs, deceased patients, and treatment histories. Circles and Xs represent ecDNAs and CGRs, respectively. The size of circles or Xs indicates the number of copy number variant segments (reference sizes shown). (**g**) Distribution of mutant (variant) allele frequencies in early, intermediate, or late mutations for RAM12.01 and RAM14006. Source data

We generated WGS data (median coverage of 23×; range, 10×–43×) from a subset of RAM tumors (one sensitive and 21 acquired- resistant; eight cases; six sites) (Supplementary Table 3). We observed no significant differences in the median SNVs or IDs based on treatment histories. The mean WGS-estimated TMB was 97 mutations per Mb (Fig. 3a), which is higher than WES-estimated TMB (Fig. 1a) and WGS-estimated TMB (39.6 mutations per Mb) in earlier-stage, MAPKi/ICB-naive CM (Supplementary Table 7). We identified a mean of 493 SVs per tumor (range, 254–1,708) (Fig. 3a), in contrast to a mean of 342 or 106 in mucosal or CM cohorts, respectively^4^. We first classified SV/rearrangements as clustered or non-clustered^24,25^ and found 67% as non-clustered translocations, 17% as non-clustered deletions and 13% as non-clustered inversions. Rearrangement signature (RS) 2 (ref. ^24^), defined by large (>100 kb) non-clustered deletions, inversions and inter-chromosomal translocations, was most frequent, regardless of case, site or treatment (Fig. 3b). To dissect the temporality of RSs, we focused on two RAM cases with multiple tumors to reconstruct the phylogeny and determine early (Fig. 3c and Extended Data Fig. 4a), intermediate (Fig. 3c) and late SVs (Fig. 3c and Extended Data Fig. 4a). Analysis of RS1 to RS6 revealed that (1) all six RSs occurred at ≥4% in every tumor among early SVs; (2) the frequencies of RS4 or RS5 (characterized by <100-kb deletions and enriched in *BRCA*1/2-deficient breast tumors^26^) increased among intermediate (versus early) SVs; and (3) the frequencies of RS2 increased among late SVs (Fig. 3c and Extended Data Fig. 4a). Moreover, analyzing WGS derived from clinical (pre and post) MAPKi tumors (10 pre and 17 post from 10 patients), we observed that RS2 enrichment dominated late SVs in both pre and post tumors (Extended Data Fig. 4b).

To assess DNA double-strand break (DSB) repair mechanisms, we analyzed the breakpoint-junctional sequences of early (Fig. 3d and Extended Data Fig. 4c), intermediate (Fig. 3d) and late SVs (Fig. 3d and Extended Data Fig. 4c). Among early SVs, 76% of breakpoints displayed a homologous sequence (HS) size of 0–1 base pairs (bp), supporting non-homologous end joining (NHEJ) as a key DSB repair mechanism. Among intermediate and late SVs, we inferred either NHEJ or NHEJ + alternative NHEJ. We validated the importance of NHEJ across temporal SVs using WGS data from the clinical pre and post MAPKi cohort (Extended Data Fig. 4d). Thus, NHEJ and alternative NHEJ represent potential targets to blunt SV-driven melanoma progression.

Moreover, we identified pathway enrichments of genes overlapping somatic SVs. First, SV-related deletions occurred in *CDKN2A* (52%), *PTEN* (38%) and *BRCA*1 (~29%) (Fig. 3e). Notably, SVs in chr6p spanning HLA class I and II genes were highly recurrent (62%) (Fig. 3e). Second, we identified recurrent translocations (≥4/21 tumors) involving oncogenes such as *PDGFB* and *JAZF1* (Supplementary Table 11). Third, 491 somatic SV-related genes (Supplementary Table 12) recurrently (>50%) and significantly enriched for immune pathways, such as antigen processing and presentation, IFN-γ, complement and IL-6-mediated JAK-STAT3 signaling (Fig. 3f). Fourth, a higher number of recurrent translocations associated with MAPKi-only or MAPKi+ICB treatment histories (Fig. 3g). MAPKi-only tumors harbored recurrent (60%) duplications of *HOXA* family genes and *ETV1*. MAPKi+ICB tumors harbored frequent (43%) SVs involving *SMAD4*. Notably, MAPKi-only or MAPKi+ICB tumors harbored recurrent deletions in MMR genes (*MSH2*, *MSH6* and *MLH1*). Finally, we identified *BRAF* amplification via intrachromosomal complex genomic rearrangements and extrachromosomal DNAs in association with MAPKi treatment (Fig. 3h and Extended Data Fig. 4e,f).

Among ID signatures, ID1 and ID2 were most frequent (Fig. 3i), which suggests mutagenesis via slippage of replicated DNA strands. SBS signatures of defective MMR (SBS6, SBS15, SBS20 and SBS26) were prevalent (Fig. 3i). Their proportions were significantly (Kruskal–Wallis test, *P* = 0.0082) higher with MAPKi-only and MAPKi+ICB treatments (Fig. 3j). We then temporally ordered WGS-based somatic SNVs. The average mutant allele frequencies of early, intermediate and late SNVs were, respectively, 0.47, 0.41 and 0.20 (Extended Data Fig. 4g). Consistent with WES-based findings (Fig. 2b), UV-related SBS signatures dominated early SNVs (Fig. 3k). Intermediate SNVs enriched for SBS10b (mutations in the polymerase epsilon exonuclease domain, which are associated with hypermutations >100 per Mb) (Fig. 3k). Late mutations enriched for SBS20 and SBS26 (defective MMR) and SBS3 (defective HRR) (Fig. 3k). Moreover, we identified somatic SNVs (3/21 intermediate, 2/21 late) in the *TERT* promoter (−124C/T and −146C/T). Finally, we assessed the temporality of HRR and MMR alterations. Early nonsense *BRCA2* mutations occurred in RAM19005 (ICB-only), and intermediate SV-associated *BRCA1* deletions occurred in RAM14006 (MAPKi+ICB), which may explain RS5 enrichment (Fig. 3c). MMR gene deletions occurred as early, intermediate and late somatic SVs, and somatic non-synonymous SNVs occurred late in MMR genes (*MLH3*, *MSH6*, *MSH2* and *PMS2*), potentially contributing to MMR SBS signatures (Fig. 3k).

### Organ site-specific transcriptomic features

**Fig. 4:**
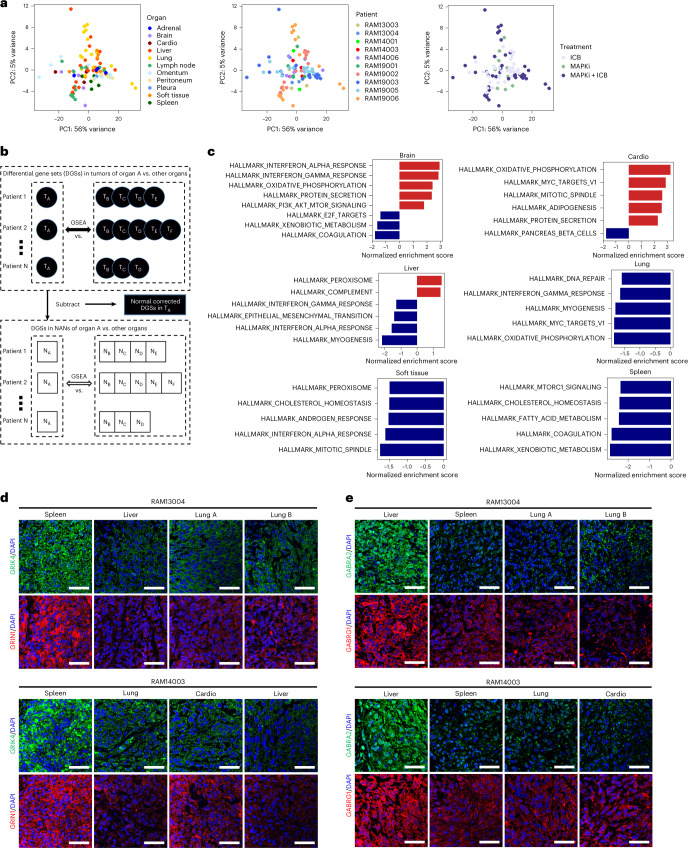
Normal tissue-depleted transcriptomic signatures of organ-specific metastases. **a**, PCA of transcriptional profiles of RAM tumors (*n* = 93) colored by organ sites, RAM cases and treatment histories. **b**, DGS analyses of tumor-cell-enriched, organ-specific transcriptomes. DGSs between tumors of organ A versus other organs (that is, B, C, D, E and F) are identified by GSEA. DGSs are similarly identified between NAN tissues (*n* = 67) of organ A versus other organs. Subtraction of the latter from the former identifies normal-corrected/depleted (or tumor-cell-enriched) DGSs of organ-specific transcriptomes. **c**, Normalized enrichment scores (NESs) of top five positively and negatively enriched gene sets (hallmark gene sets, Molecular Signatures Database) in organ-specific metastases of therapy-resistant cutaneous melanoma from rapid autopsies. **d**,**e**, Immunofluorescent staining of indicated proteins within tumor tissues of distinct organs from two RAM cases (ruler, 50 μm; experiment performed once). PC, principal component. Source data

**Extended Data Fig. 5:**
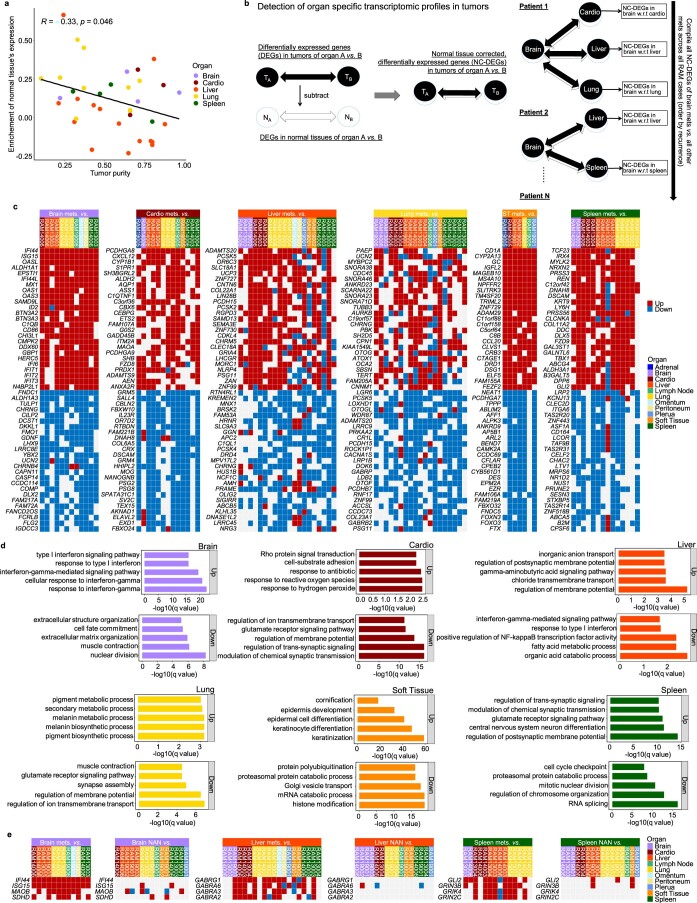
Organ-specific, normal tissue-depleted expression profiles of metastatic tumors. (**a**) Correlation between tumor purity and enrichment of normal organ-tissue expression in tumor samples (n = 36) across the brain, cardio, liver, lung and splenic metastases. Only tumor samples with both WES and RNA-seq data available were included in this analysis. *P* value, two-sided Pearson correlation. (**b**) Differentially expressed gene (DEG) analyses of multi-organ metastases. DEGs between tumors from two organs are corrected by removing DEGs between non-adjacent normal tissues from the two respective organs. In the example of the brain, normal corrected-DEGs (NC-DEGs) are identified between brain metastases with respect to (w.r.t.) tumors from all other available organ sites in the same RAM case (patient). Recurrence of organ-specific, NC-DEGs is computed by combining the NC-DEGs of brain metastases across all available cases or deceased patients. (**c**) Top 25 upregulated (red) and downregulated (blue) genes in tumors metastatic to one specific organ (brain, cardio, liver, lung, soft tissue, or spleen; displayed horizontally) versus other organs (displayed vertically). (**d**) Top 5 significantly enriched gene ontology (GO) terms (biological processes) for the recurrently upregulated or downregulated genes in tumors metastatic to one specific organ versus other organs. Recurrently upregulated or downregulated genes, ≥ 30% of comparisons (in c). (**e**) Recurrence of tumor enriched, OXPHOS, IFN-induced (in brain) and neural-related genes (in liver and spleen) in case-matched, cross-organ comparisons for both tumor and non-adjacent normal tissue compartments. Source data

Consistent with ‘contamination’ of bulk tumors by organ-specific cell types, we observed an inverse correlation between WES-based tumor cell purities and enrichment of NAN gene expression (Extended Data Fig. 5a). However, bulk tumor transcriptomes did not segregate by cases, treatment histories or sites, possibly because of wide-ranging tumor purities (<25% to 90%) (Fig. 4a and Supplementary Table 2). We then devised a strategy to identify tumor-cell-enriched signatures by detecting differential gene sets (DGSs) and differentially expressed genes (DEGs), where DGSs and DEGs of organ-specific metastasis are (1) depleted bioinformatically of NAN DGSs and DEGs across case-matched, cross-organ, pair-wise comparisons and (2) consistent across multiple such comparisons in ≥3 cases (Fig. 4b and Extended Data Fig. 5b). We observed upregulated and downregulated DGSs and DEGs specific to brain, cardiac, liver, splenic, lung and ST metastases (Fig. 4c and Extended Data Fig. 5c). We also performed Gene Ontology (GO) enrichment analysis of recurrent DEGs (Extended Data Fig. 5d). Brain metastases organ-specifically upregulated IFN response signatures and associated with oxidative phosphorylation and PI3K-AKT-mTOR signaling^27,28^. Cardiac metastases upregulated oxidative phosphorylation and response to reactive oxygen species and downregulated neurotransmitter and anabolic hypoxia genes. Liver and splenic metastases both upregulated neural genes/pathways. Liver metastases upregulated the complement pathway but downregulated IFN response genes. Lung metastases upregulated snoRNAs and pigment biosynthesis, whereas ST metastases upregulated epidermal differentiation genes. We confirmed that organ-specific, tumor-cell-enriched transcripts were not differentially expressed by corresponding NANs (Extended Data Fig. 5e). At the protein level, we validated the glutaminergic versus the GABAnergic phenotypes of splenic (Fig. 4d) versus liver (Fig. 4e) metastases.

### Organ-specific tumor microenvironmental–macroenvironmental interactions

**Fig. 5:**
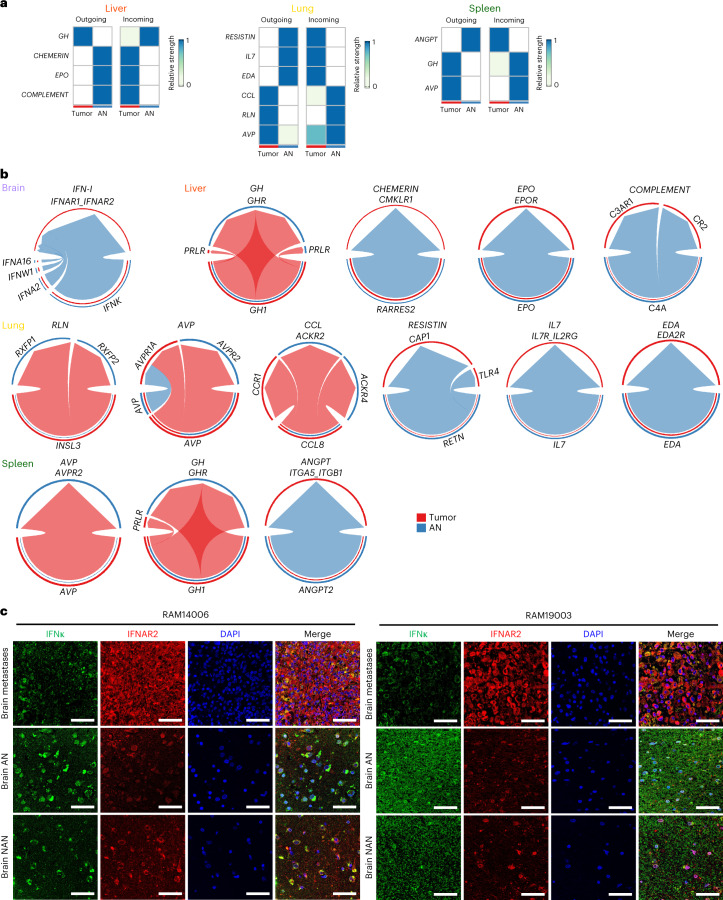
Organ-specific ligand–receptor signaling between metastatic tumors and tumor-adjacent tissue macroenvironments. **a**, Analysis of bulk RNA-seq showing signaling strengths of incoming and/or outgoing ligand–receptor pairs between tumor and AN (also known as macroenvironmental) tissues in the liver, lung and spleen. Values are row-scaled. **b**, As in **a**, except data visualized as chord diagrams and included the brain. Arrows point to the receivers (receptors). **c**, Immunofluorescent co-staining of IFNκ and IFNAR2 in RAM case-matched brain tumors, AN and NAN tissues (ruler, 50 μm; experiment performed once). Source data

Next, we searched for ligand–receptor signaling across metastases and AN tissues. In four tumor–AN pairs from ≥3 cases, we observed growth factor, inflammation and fibrosis ligand–receptor pairs in the brain, lung, liver and spleen, visualized as outgoing or incoming (Fig. 5a) and with directionality plus connectivity strength (Fig. 5b). Lungtumor-outgoing ligand *CCL* connected to the tumor and AN as incoming signals via the receptors *CCR1* and *ACKR2/4*, respectively (Fig. 5b). In brain metastases, AN-outgoing type I *IFN* signaled into tumors via *IFNAR1/2* (Fig. 5b), which is consistent with *IFN* upregulation by brain metastases (Fig. 4 and Extended Data Fig. 5). In lung metastases, AN-to-tumor signals consisted of *RESISTIN*, *IL-7* and *EDA*; tumor-to-AN signals consisted of *CCL*, *RLN* and *AVP*. In liver metastases, AN-outgoing ligands included complement genes (*C4A*) (Fig. 4c), *EPO* and *CHEMERIN*, and tumor-outgoing ligands included only *GH*. Moreover, splenic metastases featured both AN-to-tumor (*ANGPT2*) and tumor-to-AN (*GH1* and *AVP*) axes. Lastly, immunofluorescence studies confirmed IFNκ expression preferentially in the AN of brain metastases, IFNAR2 intratumorally and their overlap intratumorally (Fig. 5c).

### Organ-specific tumor immune contextures

**Fig. 6:**
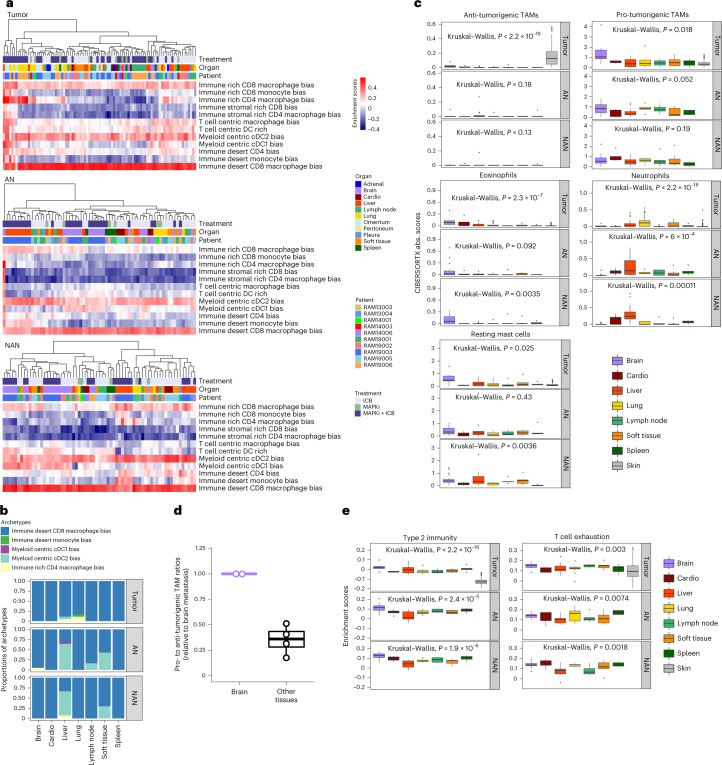
Tumor immune microenvironments and macroenvironments across organ sites. **a**, Unsupervised clustering of 12 pan-cancer immune archetypes in the RAM tumor (*n* = 93), AN (*n* = 68) and NAN (*n* = 67) tissue compartments, across RAM cases/patients, organ sites and treatment histories. Each sample’s enrichment scores of immune archetypes were used to generate heat maps. **b**, The composition of immune archetypes in the tumor, AN and NAN compartments across multiple organ sites. **c**, Absolute enrichment scores (CIBERSORTx) of anti-tumorigenic (named M1 by CIBERSORTx) or pro-tumorigenic (M2) TAMs, eosinophils, resting mast cells and neutrophils in the tumor, AN and NAN compartments of indicated organ sites, compared against the TCGA–SKCM cohort (labeled ‘skin’ as the organ site) (*n* = 344 *BRAF*^MUT^ or *NRAS*^MUT^ melanoma). Comparisons among organs by the Krustal–Wallis test. Central line of each box, median; top and bottom edges of each box, first and third quartiles; whiskers extend 1.5× the interquartile range beyond box edges. Tumor compartment (brain, *n* = 6; cardio, *n* = 4; liver, *n* = 18; lung, *n* = 17; lymph node, *n* = 13; spleen, *n* = 5; soft tissue, *n* = 10; skin, *n* = 344). AN (brain, *n* = 21; cardio, *n* = 5; liver, *n* = 17; lung, *n* = 6; lymph node, *n* = 6; spleen, *n* = 5; soft tissue, *n* = 7). NAN (brain, *n* = 20; cardio, *n* = 5; liver, *n* = 15; lung, *n* = 5; lymph node, *n* = 6; spleen, *n* = 5; soft tissue, *n* = 10). **d**, Quantifications by multiplex immunofluorescence of the ratios of pro-tumorigenic (CD68^+^CD163^+^CD206^+/−^) to anti-tumorigenic (CD68^+^iNOS^+/−^CD206^–^CD163^−^) TAMs in metastatic tumors to visceral organs (*n* = 4; RAM19003: liver, omentum; RAM19006: lung and LN) relative to ratios in tumors to the brain (*n* = 2) in two RAM cases. Box plot as defined in **c**. **e**, As in **c**, except enrichment scores of T cell exhaustion and type 2 immunity signatures in the tumor, AN and NAN compartments across organ sites. Source data

**Extended Data Fig. 6:**
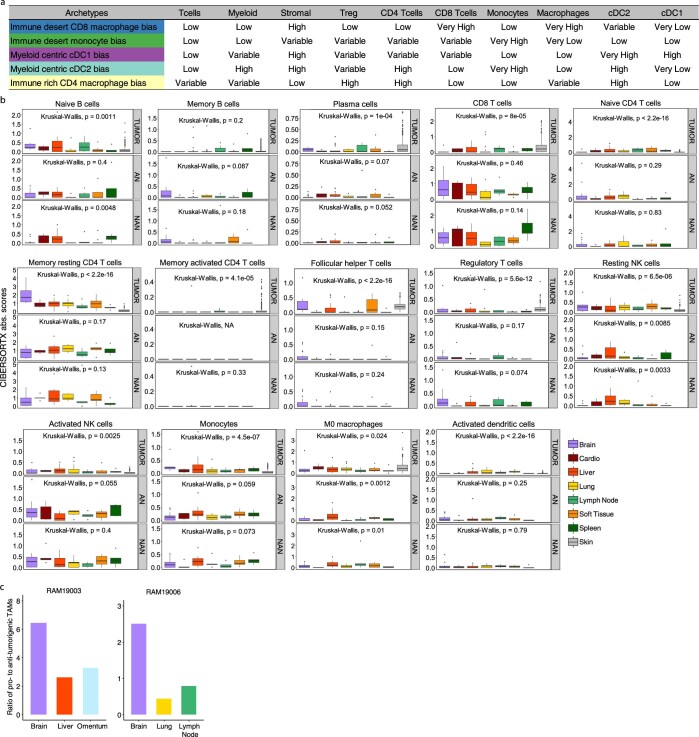
Tumor immune microenvironments and macroenvironments across multiple organs. (**a**) Summary of characteristics and immune cell features of immune archetypes identified in the RAM tissue cohort. (**b**) Absolute enrichment scores of 14 immune cell types in the tumor, adjacent normal (AN), and non-adjacent normal (NAN) tissue compartments. Comparisons among organ sites by the Kruskal-Wallis test. Central line of each box, median; top and bottom edges of each box, first and third quartiles; whiskers extend 1.5x the inter-quartile range beyond box edges. TCGA–SKCM labeled as ‘skin’ in the tumor compartment category (n = 344 *BRAF*^MUT^ or *NRAS*^MUT^ melanoma). Tumor compartment (brain, n = 6; cardio, n = 4; liver, n = 18; lung, n = 17; lymph node, n = 13; spleen, n = 5; soft tissue, n = 10, skin, n = 344). AN (brain, n = 21; cardio, n = 5; liver, n = 17; lung, n = 6; lymph node, n = 6; spleen, n = 5; soft tissue, n = 7). NAN (brain, n = 20; cardio, n = 5; liver, n = 15; lung, n = 5; lymph node, n = 6; spleen, n = 5; soft tissue, n = 10). (**c**) Quantifications by multiplex immunofluorescence of the ratios of pro-tumorigenic (CD68^+^CD163^+^CD206^+/−^) to anti-tumorigenic (CD68^+^iNOS^+/−^CD206^−^CD163^−^) TAMs in metastatic tumors to the brain and to indicated visceral organs in two RAM cases. Source data

We next evaluated organ-specific (intra)tumor immune microenvironments (TIMEs) and immune macroenvironments (within AN and NAN tissues). Analysis of 12 pan-cancer immune archetypes^29^ via unsupervised clustering revealed immune macroenvironments, more than TIMEs, as organ-specific or patient-specific (Fig. 6a). We then assigned each tissue sample to the highest enrichment-scoring immune archetype and calculated the distribution of archetypes for each organ (Fig. 6b and Extended Data Fig. 6a). AN and NAN immune archetypes were similar in each organ, and TIMEs uniformly (~100%) enriched for the immune-desert, CD8^+^-macrophage-biased archetype linked to T-cell exhaustion and the worst survival in patients from the TCGA–SKCM cohort^29^. This contrasted with a lower frequency (~60%) of the same archetype in TCGA–SKCM tumors^29^, 30% of which belong to two immune archetypes: T cell-centric macrophage biased and T cell-centric dendritic cell biased^29^. These and the immune-rich CD8^+^ or CD4^+^ archetypes were all but absent in our RAM cohort. Interestingly, ~5% of liver TIMEs comprised the myeloid-centric, cDC2-biased archetype (associated with tumor fibrosis).

Using CIBERSORTx, we evaluated RAM versus TCGA–SKCM immune contextures (Fig. 6c and Extended Data Fig. 6b). Across 22 immune cell types, we observed consistent patterns between AN and NAN tissues, except for plasma cells, regulatory T cells and naive/memory B cells. Relative to RAM tumors, TCGA melanomas displayed a higher proportion of anti-tumorigenic macrophages (Fig. 6c). Across RAM organs, brain metastases enriched for pro-tumorigenic macrophages, eosinophils and resting mast cells. Using multi-spectral immunofluorescence, we confirmed a higher pro- to anti-tumorigenic tumor-associated macrophage (TAM) ratio in brain metastases (Fig. 6d and Extended Data Fig. 6c). In addition, lung metastases preferentially comprised neutrophils, potentially related to tumor-derived *CCL8* (Fig. 5). Finally, RAM (versus TCGA–SKCM) enriched for T-cell exhaustion and type-2 immunity (Fig. 6e).

## Discussion

This RAM study begins to build foundational insights into highly evolved and lethal CMs that resist MAPKi and/or ICB therapies. By comparative analysis of acquired-resistant CM (preceded by only one of the two types of therapies) with patient-matched pre-treatment tumors, we resolved further how each therapy distinctly and convergently shapes the high mutational, CNA and SV burdens of acquired-resistant CM. SMGs and genes altered by CNAs and SVs enrich in immune-evasive processes (for example, *BRAF*^MUT^ amplification and loss-of-function alterations in *B2M*, *JAK2*, *CD274*/*PD-L1* and *PTEN*; Supplementary Table 13) that may confer cross-therapy resistance, accelerating lethal disease progression. Notably, evolution of MAPKi (versus ICB) resistance shifts the mutational signatures, implicating therapy-elicited DNA damage and/or deficiency in repair pathways (for example, MMR, BER and HRR) as culprits. The evolution of late/private SVs, regardless of treatment history, enriches for RS2. Analysis of breakpoint-junctional sequences of SVs suggests NHEJ as a MAPKi or ICB co-target. Overall, multiple forms of genomic instability may cause and/or result from resistance evolution, with therapeutic implications that warrant mechanistic studies.

Acquired therapy resistance co-evolves with, and may also promote, metastatic progression. However, comparative analysis of RAM versus earlier-stage and MAPKi/ICB-naive CM cohorts is limited by cross-study technical variables (sequencing depth, read lengths, tumor purities and library preparation) and by RAM’s relatively small sample size, which may contribute to false-positive SMGs. We mitigated false positives by requiring SMGs to display gene expression and by analyzing validation cohorts. Future RAM studies should expand the current cohort size and increase representation of CM subtypes, ethnic and ancestral diversities and the treatment-naive landscape.

Our analysis, by computationally depleting bulk metastatic tumor transcriptomes of patient-matched and organ-matched normal tissue-derived transcriptomes, sheds light on organ-specific metastatic signatures. Liver and spleen metastases display neural differentiation, suggesting therapeutic targets^30,31^. Melanoma brain metastasis (MBM) displays signatures of IFN signaling, oxidative phosphorylation and PI3K-AKT signaling^27,28,32^. The brain-specific macroenvironment appears to be a predominant source of IFN ligands. Overall, RAM tumors, including MBM, strongly display an immune-desert but CD8^+^-macrophage-biased archetype with enrichment of T-cell exhaustion. For MBM, loss of antigen presentation and enrichment of type-2 immunity suggest TGFβ blockade and upregulation of cytotoxic natural killer (NK) cell-mediated or CD4^+^ T cell-mediated anti-tumor immunity^33^ as potential therapeutic strategies. The pro-tumorigenic TAM phenotype of MBM also suggests therapeutic co-targets. Thus, we have uncovered a preliminary set of organ-specific metastatic signatures, tumor macroenvironment crosstalks and immune contextures that characterize therapy-resistant CM, justifying expanded RAM-based and functional analyses.

## Methods

### Rapid autopsies and samples

We performed warm autopsies with informed consent at the University of North Carolina at Chapel Hill and the University of California, Los Angeles (UCLA). In brief, patients or persons holding the healthcare power of attorney signed the Autopsy Authorization Form and the institution-specific tumor tissue procurement and banking consent form. Research in this study involving autopsy specimens does not meet the regulatory definition of human subject research. The last tissue sample of each case was excised and stored no longer than 6 hours from death. We collected metastatic tumor, AN (≤1 cm away from tumor border) and NAN (>1 cm) tissues from 11 RAM cases. We collected formalin-fixed, paraffin-embedded (FFPE) tissues from all available sites. If tissue was sufficient, we also collected snap-frozen tissues and stored them at −80 °C. An autopsy pathologist (Leigh B. Thorne) reviewed hematoxylin and eosin tissue sections. Histopathologic analysis selected for high tumor content and against necrosis, AN with ≤10% tumor cell contamination and NAN without tumor cell contamination. We further selected for tumor purity >30% based on Sanger-sequencing-estimated *BRAF* or *NRAS* mutant allele frequencies (*BRAF* forward primer, 5′-GACTCTAAGAGGAAAGATGAAGTAC-3′; *BRAF* reverse primer, 5′-GTTGAGACCTTCAATGACTTTCTAG-3′; *NRAS* forward primer, 5′-GGCTTGAATAGTTAGATGCTTATTTAACCTTGGC-3′; and *NRAS* reverse primer, 5′-GCTCTATCTTCCCTAGTGTGGTAACCTC-3′).

### Clinical samples

Tumor tissues from living patients with CM and patient-matched normal tissues were collected with the approval of institutional review boards at UCLA and Vanderbilt Ingram Cancer Center and with informed consent of each patient or the patient’s legal representative. We analyzed by WES 88 tumor samples from 28 patients at UCLA with *BRAF*^MUT^ CM obtained before treatments with and responses to MAPKi and then at the time of disease progression (that is, acquired resistance), along with patient-matched normal tissues (Supplementary Table 5). From PDX models collected from patients at UCLA, we analyzed by WES 29 tumors and patient-matched normal tissues (from eight patients with *BRAF*^MUT^ or *NRAS*^MUT^ CM) treated with vehicle or trametinib (at sufficient in vivo doses to induce tumor regression) in NSG mice (Supplementary Table 6). Also, we analyzed by WES 14 tumor samples from seven patients with CM at Vanderbilt obtained before treatments with and responses to ICB and then at the time of disease progression (Supplementary Table 5). Sex/gender was self-reported and not considered in the study design given that each cohort size was small. Participation in research was not compensated.

### PDXs and treatments

To develop PDX models, tumor fragments derived from metastatic CM, with approval by UCLA institutional review boards and the UCLA Animal Research Committee, were transplanted subcutaneously in sex-matched NSG mice (4–6 weeks old) from the UCLA vivarium or Jackson Laboratory. We conducted all animal experiments in accordance with approved protocol and regulations (ARC 2016-086). We adhered to the maximal tumor size for experimental endpoints, which was ~1,500–2,000 mm^3^ without mobility impairment and with body condition score >2. We implanted one tumor fragment in each mouse on the flank and measured tumors with a calliper every 2 days. Tumor volumes were calculated by (length × width^2^)/2, and we excluded data from occasional animals that died before final analysis. We assigned tumors with volumes ~500 mm^3^ randomly into experimental groups. A special mouse chow (Test Diet) incorporated trametinib (LC Laboratories) to achieve daily dosing at 5 mg/kg/day.

### WES and WGS

We extracted gDNAs from snap-frozen tumors, NANs and ANs using the QIAGEN AllPrep DNA/RNA Mini Kit; from FFPE tumors (clinical ICB cohort) using the QIAGEN QIAamp DNA FFPE Tissue Kit; and from frozen blood using the QIAGEN FlexiGene DNA Kit. Quantification of gDNAs was based on NanoDrop (Thermo Fisher Scientific) and/or Qubit fluorometer using the Qubit dsDNA Broad Range (BR) Assay Kit (Life Technologies). Size and quality of gDNAs were based on TapeStation (Agilent Technologies) and/or agarose gel electrophoresis to ensure gDNA library preparation from equal gDNA input and from intact high-molecular-weight gDNA. We prepared whole-exome libraries using the Roche NimbleGen Exon-Seq Kit, the Roche NimbleGen SeqCap Kit or the Roche KAPA HyperPlus Library Preparation Kit with KAPA HyperCap Workflow version 3.0 for exome hybridization (Supplementary Table 14) and whole-genome libraries using the Roche KAPA HyperPrep Kit. In brief, after enzymatic fragmentation of gDNAs, the libraries were constructed by end-repairing and A-tailing the fragmented DNAs, ligation of adapters and polymerase chain reaction (PCR) amplification. After library construction, we quantified indexed libraries for equal molar pooling and paired-end sequenced with a read length of 2 × 150 bp on the Illumina HiSeq 3000 or Illumina NovaSeq 6000 S4 platform.

### RNA-seq

We extracted total RNAs from snap-frozen tissues using the QIAGEN AllPrep DNA/RNA Mini Kit and the Ambion mirVana miRNA Isolation Kit. Total RNA quantification was based on the Qubit RNA High Sensitivity (HS) Assay Kit (Thermo Fisher Scientific) and/or a NanoDrop (Thermo Fisher Scientific). RNA quality was based on TapeStation (Agilent Technologies) and used to calculate the input for RNA library preparation. We prepared RNA libraries using KAPA RNA HyperPrep Kit with RiboErase (Roche) or TruSeq RNA Exome Kit (Illumina), TruSeq RNA Single Indexes Set A Kit (Illumina) and TruSeq RNA Single Indexes Set B Kit (Illumina) following the manufacturers’ protocols (Supplementary Table 15). In brief, RNA libraries, prepared by the KAPA RNA HyperPrep Kit with RiboErase, used 1 μg of total RNA captured by magnetic oligo-dT beads. RNAs were fragmented and cDNAs synthesized using random priming for the first strand. The second strand was synthesized (with dUTP) and marked to convert the cDNA:RNA hybrid to double-stranded cDNA (dscDNA). Then, dscDNA libraries were constructed by adding dAMP to the 3′ ends during A-tailing, and dsDNA adapters with 3′ dTMP overhangs were ligated to library insert fragments during adapter ligation. We carried out amplification of library fragments carrying appropriate adapter sequences at both ends using high-fidelity, low-bias PCR amplification, whereas the strand marked with dUTP was not amplified, to enable strand-specific sequencing. After library construction, we quantified indexed libraries for equal molar pooling and single-end sequenced with a read length of 1 × 50 bp on the Illumina HiSeq 3000 or Illumina NovaSeq 6000 S4 platform. RNA libraries prepared by the TruSeq RNA Exome Kit used 50 ng of RNA for high-quality RNA (DV_200_ > 70%) and 100 ng of RNA for medium-quality and low-quality RNA (DV_200_ < 70%). After RNA fragmentation and cDNAs synthesis, we purified dscDNAs using AMPure XP beads (Beckman Coulter). After A-tailed enrichment, we quantified cDNA libraries by the Qubit dsDNA HS Assay Kits (Thermo Fisher Scientific); quality was assessed by TapeStation. We used 200 ng of each DNA library for exome enrichment. After amplification and purification, we removed free adapters by using Illumina Free Adapter Blocking Reagent (Illumina). All libraries were pooled at equal molar and sequenced with a 2 × 150-bp read length on the Illumina NovaSeq 6000 S4 platform.

### Somatic SNVs and CNAs

We used BWA for mapping and Picard for removal of duplications. We identified somatic SNVs and IDs of tumors^34,35^ by using patient-matched normal tissues for germline reference. We called SNVs using the Unified Genotyper tool of GATK, MuTect and VarScan2 and IDs using GATK-UGF, SomaticIndelDetector of GATK (IndelLocator) and VarScan2 (calls made by at least two of three algorithms). SNV/IDs were supported by at least five reads in the tumor samples and none in the patient-matched normal tissues. Somatic SNV/IDs were then annotated by Oncotator^36^. Finally, we used Sequenza^37^ to detect tumor purity, ploidy, somatic CNAs and loss-of-heterozygosity regions.

### Significant CNA genes in RAM tumors

We applied GISTIC2.0 (ref. ^38^) to identify the significantly deleted and amplified regions using each RAM tumor’s copy number segmentation file as the input. We generated circos plots with both *q* values and G-scores representing the amplitude of the aberration. Only regions with *q* values less than 0.05 were defined as significantly altered regions. We performed Fisher’s exact test to identify differentially amplified or deleted genes in the RAM tumor cohort versus the *BRAF* or *NRAS* mutated TCGA–SKCM cohort (*n* = 345 tumors: seven stage 0, 65 stage 1, 100 stage 2, 130 stage 3, 17 stage 4 and 26 unknown). We downloaded TCGA–SKCM CNA data from cBioPortal and compiled the frequencies of CNA genes (Supplementary Table 16). We applied Fisher’s exact test to individual-level amplified or deleted events, which were counted in a given RAM case if they were identified from at least one tumor of that case. We identified RAM frequency-enriched (versus TCGA–SKCM) CNA genes as amplified or deleted genes with a false discovery rate (FDR)-adjusted *P* < 0.05 and a higher frequency in RAM (versus TCGA–SKCM) cohort.

### CNA genes enriched in post (versus pre) acquired MAPKi resistance

We compared the patient-level frequency of each amplified or deleted gene in post-treatment (acquired-resistant) versus pre-treatment melanoma by using Fisher’s exact test. When multiple post tumors were available from a given patient, they were considered as an entirety, and an amplified or deleted gene was counted in this patient if it was identified from at least one of the post tumors. Multiple pre-treatment tumors from one patient were also counted as an entirety. We nominated a post-enriched amplified or deleted gene if its frequency is higher in post (versus pre) tumors and the FDR-adjusted *P* value is less than 0.05.

### CNA genes enriched in post (versus pre) acquired ICB resistance

An amplified or deleted gene enriched in acquired ICB resistance was defined by its amplification or deletion frequency in the post tumors being >2× that in the pre tumors and by its detection in ≥2 patients’ post tumors.

### SMGs

We applied MutSig2CV to identify SMGs using each RAM case’s mutational profile as the input and each RAM case (not each tumor sample) as an identifier. A mutation exists in a given RAM case if it was identified in ≥1 tumor. To circumvent the limitation of a small sample size, we inflated type I error by not performing multiple testing^39^ and identified by MutSig2CV genes at *P* < 0.05. To reduce false positives, we nominated SMGs by filtering for RNA expression. Based on the mean values of normalized expression levels (log_2_ counts per million (CPM)) of RAM tumors, we annotated MutSig2CV SMGs as no expression (mean log_2_CPM < 0), expressed (0 ≤ mean log_2_CPM < 4) or highly expressed (mean log_2_CPM > 4) and excluded those with no expression. We performed Fisher’s exact test to compare the patient-level frequency of each SMG in the RAM versus TCGA–SKCM cohorts and used the FDR approach to adjust the *P* values. We then performed GO enrichment analysis with the clusterProfiler package to detect the significant biological processes for expressed SMGs. Moreover, we identified SMGs with MutSig2CV for post tumors from patients with MAPKi-only or ICB-only treatments by using each patient’s mutational profile of post tumor(s) as input and each patient (not each post tumor) as an identifier. For patients with multiple post tumors, a mutation was considered to exist in this patient if it was detected in ≥1 tumor. We defined post/acquired-resistant SMGs as genes identified by MutSig2CV at *P* < 0.05 in the post tumors and not identified in the patient-matched pre tumor(s).

### Phylogeny and mutational signatures

We performed phylogenetic analysis using the PHYLIP program with the parsimony algorithm^35^ and annotated each tree with potential drivers of tumorigenesis and/or resistance. All tumors in each case shared early somatic SNVs; a subset shared intermediate SNVs; and late SNVs were unique to one tumor. The contribution of each mutational type and mutational signature was then determined for early, intermediate and late mutations through the R package deconstructSigs^40^ with COSMIC SBS signatures version 3.3 as reference. For the pre and post MAPKi-only and ICB-only cohorts, we identified mutations unique to post tumors per patient, which were then subjected to mutational signature analysis. We used the R package MutationalPatterns with COSMIC ID signatures version 3.3 as reference to determine ID signatures underlying all identified *B2M* IDs.

### ITH and preferentially mutated genes

We conducted a subclonal analysis for each RAM case using PyClone-VI^41^ and assessed each mutation’s cancer cell fraction (CCF). Mutations were clonal if the CCF approaches 1; otherwise, mutations were subclonal. The ratio of subclonal mutations to all mutations determined ITH. We determined preferentially mutated genes for each organ as follows: (1) selected for non-synonymous mutations; (2) calculated ΔCCF (see formula in Extended Data Fig. 3b) of each mutation in the tumor of one specific organ (that is, T_A_) versus tumors of other organs (that is, T_B_…T_N_); (3) identified organ-specific enriched mutations with a ΔCCF of >0.2 for each RAM case; and (4) defined a gene as ‘preferentially mutated genes’ of organ A when organ-A-enriched mutations in this gene occurred in ≥3 patients.

### WGS-based SV analysis

We mapped WGS reads to GRCh38/hg38 human reference genome using BWA-MEM^42^. SAMtools^43^ sorted alignments and removed PCR duplicates. CNVkit^44^ with default parameters called CNVs. SVs were concordant by two or more of three methods: SvABA^45^, TIDDIT^46^ and DELLY^47^. For high-coverage data (>15×), we used default parameters. The parameter minimum number of points (-l) was set to 5 in TIDDIT for low-coverage data. SVs detected in matched-normal genomes were removed from the tumor samples to infer somatic SVs. We estimated rearrangement signatures based on a previous classification^26^. For RAM cases with ≥2 tumors, we computed early, intermediate (only feasible with RAM12.01 and RAM14006) and late somatic SVs. For each case, early and late somatic SVs consisted of SVs common to all tumors and unique to each tumor, respectively, and the rest were classified as intermediate. We carried out breakpoint junctional sequence analysis by examining the presence of homologous sequences. Homologous sequences with 0–1 bp, 2–6 bp and >6 bp were attributed to NHEJ, alternative NHEJ (microhomologous end joining) and HRR, respectively. Genes overlapping with somatic SVs were annotated using AnnotSV^48^. We performed pathway enrichment analysis using the Molecular Signatures Database (MSigDB)^49^ with pathway datasets listed in KEGG, Reactome and Pathway Interaction databases.

### Reconstruction of focal amplifications

Focal amplicon identification and elucidation of circular extrachromosomal DNAs (ecDNAs) and complex genomic rearrangements (CGRs) using WGS data were carried out by AmpliconArchitect^50^. In brief, we determined the list of potential intervals for each amplicon, for which copy numbers and SVs were estimated using read depth and discordant read signatures. It then constructed a breakpoint graph. Simple cycles were then decomposed from the breakpoint graphs and amplicons classified into ecDNAs, CGRs and linear amplicons. We used CNVKit to infer the initial set of CNV seed regions. SV view of amplicons was generated using functions available in AmpliconArchitect.

### WGS-based mutation analysis

Somatic SNVs and IDs were identified using Strelka2 (ref. ^51^) with default parameters and then subjected to mutational and ID signature analyses using the R package deconstructSigs^40^ and MutationalPatterns^52^, respectively, with COSMIC SBS/ID signatures version 3.3 as reference. Classification of early, intermediate and late somatic SNVs was in accordance with that for WES-based mutations.

### RNA-seq analysis

We analyzed single-end and paired-end RNA-seq data^3,35^ by mapping transcriptome reads to the GRCh38/hg38 human reference genome using HISAT2. Gene-level counts were estimated by the htseqcount program. The normalized expression level of each gene, log_2_CPM, was calculated by the R package edgeR^53^ and batch corrected with the ‘removeBatchEffect’ function in the limma R package^54^. We performed principal component analysis (PCA) using the prcomp function in R package stats to visualize the clustering of samples.

### Organ tissue expression in RAM tumors

We first used the DESeq2 package to detect DEGs between NANs from one specific organ versus other organs. We defined organ-specific normal gene signatures as the top five significantly upregulated genes, all of which were confirmed to display organ-specific expression in the human protein atlas^55^. For RAM tumors, we performed single-sample gene set enrichment analysis (GSEA) to generate the enrichment scores of the organ-matched normal organ-specific gene signature. CPM values were input into the gene set variation analysis (GSVA) program using the default ‘kcdf=Gaussian’ option.

### DGS analysis

GSEA via the fgsea package used the human hallmark (H) gene sets (MSigDB). Genes were pre-ordered by the log_2_-transformed expression fold change metrics (log_2_FC). We calculated the enrichment nominal *P* values by permutation test (100,000 permutations), with Benjamini–Hochberg FDR correction for multiple testing. We then performed GSEA to identify the DGSs for both tumors and NANs of one organ versus other organs. We defined normal-corrected DGSs (NC-DGSs) in tumors of organ A as those DGSs detected in the tumors of organ A (versus other organs) but not in the NAN compartment of organ A (versus other organs).

### DEG analysis

To filter out normal tissue-specific DEGs from bulk tumor transcriptomes, we used RNA-seq derived from each tumor’s organ-matched NANs and public datasets of normal organ/tissue gene expressions from the Illumina Human Body Map and GTEx. DEGs between a tumor pair from two organs (for example, T_A_ versus T_B_) were corrected for expression of the same genes between the normal tissue pair of the same two organs (for example, N_A_ versus N_B_). We calculated normal-corrected fold change (NC-FC) of each gene between T_A_ versus T_B_ as the FC of this gene between T_A_ versus T_B_ divided by the FC of this gene between N_A_ versus N_B_. We defined the genes with NC-FC > 2 or NC-FC < 0.5 as the upregulated or downregulated NC-DEGs between these two organs. We then computed the recurrence (≥30%) of NC-DEGs by collecting NC-DEGs of tumors from one specific organ against all other tumors from other organs of the same RAM case across all RAM cases. GO enrichment analysis via the ‘clusterProfiler’ package^56^ identified the top five significant GO biological processes for recurrently upregulated or downregulated NC-DEGs of each organ-specific metastasis.

### Analysis of tumor–macroenvironment interactions

Only organ sites with ≥4 tumor–AN pairs and four tumor–NAN pairs available from ≥3 RAM cases were included for this analysis using the CellChat R package^57^. We culled significant ligand–receptor signaling that was detected from tumor–AN pairs of one specific organ site but not from tumor–NAN pairs of the same organ site. The netAnalysis_signalingRole_heatmap function was used for visualization. The circle plots depicting tumor–AN interactions of each organ were generated by applying netVisual_aggregate with the options layout = ‘circle’.

### Analysis of immune contextures

We performed single-sample GSEA to generate the absolute enrichment scores of the 12 immune archetypes for each tissue, using CPM values of all expressed genes as input for GSVA^58^ in the default ‘kcdf=Gaussian’ option. We then assigned each tissue to the highest enrichment scoring immune archetype. CIBERSORTx^59^ was used in the ‘absolute mode’ to estimate infiltration levels of 22 immune cell types with CPM values as input. We downloaded the gene-level normalized read counts (RSEM, file name: Batch normalized from Illumina HiSeq_RNASeqV2) of TCGA–SKCM RNA-seq (inclusive of only *BRAF* or *NRAS* mutant tumors) from cBioPortal. CIBERSORTx estimated the absolute abundance of 22 immune cell types with the normalized expression level as an input. We calculated the enrichment scores of two signatures, ‘T cell exhaustion’ and ‘type 2 immunity’^33^, by GSVA using the default ‘kcdf=Gaussian’ option.

### Immunofluorescence

FFPE RAM tissue sections were heated at 90 °C for 25 minutes and immersed in xylene and gradient ethanol to achieve deparaffinization and re-hydration. Then, tissue sections were subjected to heat at 95 °C 10 mM citrate buffer (pH 6.0) for 15 minutes to retrieve antigens. After permeabilization and blocking with 0.1% Triton X-100/10% normal goat serum in PBS for 1 hour, tissue sections were incubated with primary antibodies, including anti-GRIK4 (Invitrogen, MA5-31745, 1:200), anti-GRIN1 (Abcam, ab109182, 1:50), anti-GABRG1 (Invitrogen, PA5-99317, 1:100), anti-GABRA2 (Invitrogen, PA5-106894, 1:100), anti-IFNκ (Novus, H00056832-M01, 1:50) and anti-IFNAR2 (Invitrogen, PA5-119915, 1:200), at 4 °C overnight. Visualization was achieved with goat anti-mouse IgG highly cross-absorbed secondary antibody, Alexa Fluor Plus 488 (Invitrogen, A32723, 1:400) or goat anti-rabbit IgG highly cross-absorbed secondary antibody, Alexa Fluor 555 (Invitrogen, A-21429, 1:500), and nuclei were counterstained by DAPI (Sigma-Aldrich, D9542). We captured images on a Leica confocal SP8-STED/FLIM/FCS microscope.

### Multi-spectral immunofluorescence

Using Ventana Discovery Ultra (Roche) and Opal fluorophores (Akoya Biosciences), we deparaffinized 5-µm-thick tissue sections using EZ-Prep reagent (Roche) and retrieved antigens in CC1 buffer (pH 9, 95 °C; Roche). Discovery Inhibitor (Roche) was applied to inhibit enzymatic activities, followed by six sequential rounds of staining. Each round included the addition of a primary antibody followed by secondary antibody detection using either OmniMap anti-Ms HRP (Roche, 760-4310, ready-to-use) for mouse or OmniMap anti-Rb-HRP (Roche, 760-4311, ready-to-use) for rabbit following the manufacturer’s specifications. We amplified signals by using Opal fluorophores at 1:400. Between rounds of staining, the tissue sections underwent heat-induced epitope retrieval to remove the primary/secondary-HRP antibody complexes before staining with the subsequent antibody. The primary antibodies and corresponding fluorophores are anti-MRT-1 (Abcam, ab210546, 1:200) in Opal 480; anti-iNOS (Abcam, ab115819, 1:200) in Opal 520; anti-CD68 (Roche, 790-2931, ready-to-use) in Opal 570; anti-CD163 (Abcam, ab182422, 1:200) in Opal 620; anti-CD206 (Cell Signaling Technology, 91992S, 1:200) in Opal 690 and anti-SOX10 (Abcam, ab227680, 1:200) in Opal 780. We counterstained nuclei with Spectral DAPI (Akoya Biosciences, FP1490) and mounted the stained tissues with ProLong Diamond Antifade mounting medium (Thermo Fisher Scientific). Subsequently, we imaged stained tissues (×20) using the Vectra Polaris imaging system (Akoya Biosciences). After image capture, unmixing of the spectral libraries was performed with inForm software (Akoya Biosciences). Unmixed images were then imported into HALO (Indica Labs) for stitching, cell segmentation and cell phenotyping. We analyzed whole tumor regions from each slide. Data were exported and graphed with Prism (GraphPad). Representative images were exported from HALO after spectral unmixing.

### Statistical methods

We conducted statistical analyses in R 4.02, Python 3.8.0, Python 2.7.17 and Prism.

### Reporting Summary

Further information on research design is available in the Nature Portfolio Reporting Summary linked to this article.

## Online content

Any methods, additional references, Nature Portfolio reporting summaries, source data, extended data, supplementary information, acknowledgements, peer review information; details of author contributions and competing interests; and statements of data and code availability are available at 10.1038/s41591-023-02304-9.

## Supplementary information

Reporting Summary

Supplementary Tables Supplementary Table 1. Clinical information and tissue collection. Table 2. WES data quality, characteristics and availability of sample-matched WGS and/or RNA-seq. Table 3. WGS data quality, characteristics and availability of sample-matched WES and/or RNA-seq. Table 4. RNA-seq data quality and availability of sample-matched WES and/or WGS. Table 5. Clinical information for patient-matched tumors biopsied pre-treatment and at disease progression. Table 6. Clinical information and driver mutations for PDX models. Table 7. TMB of *BRAF*^MUT^ and *NRAS*^MUT^ melanoma from published cutaneous melanoma studies. Table 8. Significantly amplified and deleted regions and genes in the RAM tumor cohort analyzed by GISTIC2.0. Table 9. Significantly amplified and deleted genes in RAM versus TCGA–SKCM (*BRAF* and *NRAS* mutated) cohorts: results of two-sided Fisher’s exact test. *P* values adjusted by FDR. Table 10. SMGs in the RAM cohort identified by MutSig2CV without multiple testing. Table 11. Recurrent somatic translocations. Table 12. Recurrent somatic structure variation-associated genes. Table 13. Mutational status of genes associated with cross-therapy resistance among RAM cases. Table 14. Summary of WES library preparation kits. Table 15. Summary of RNA-seq library preparation kits. Table 16. CNAs of the *BRAF* and *NRAS* mutant TCGA–SKCM cohorts (downloaded from cBioProtal).

## Data Availability

The BAM files of WES, WGS and RNA-seq data are deposited in the European Genome-phenome Archive (https://www.ebi.ac.uk/ega/) with accession number EGAS00001006644. Access requires a data sharing agreement. Source data are provided with this paper.

## Source data

Source Data Fig. 1 Statistical source data.
Source Data Fig. 2 Statistical source data.
Source Data Fig. 3 Statistical source data.
Source Data Fig. 4 Statistical source data.
Source Data Fig. 5 Statistical source data.
Source Data Fig. 6 Statistical source data.
Source Data Extended Data Fig. 2 Statistical source data.
Source Data Extended Data Fig. 3 Statistical source data.
Source Data Extended Data Fig. 4 Statistical source data.
Source Data Extended Data Fig. 5 Statistical source data.
Source Data Extended Data Fig. 6 Statistical source data.
