# Supplementary material for: Multi-organ landscape of therapy-resistant melanoma
Source: Nat Med. 2023 Apr 27;29(5):1123–34. doi: 10.1038/s41591-023-02304-9 (PMC10202813; doi:10.1038/s41591-023-02304-9)
Supplement: Supplementary file 1 — Reporting Summary [file 41591_2023_2304_MOESM1_ESM.pdf]

nature portfolio

Corresponding author(s): Roger Lo

Last updated by author(s): Mar 11, 2023

Reporting Summary

Nature Portfolio wishes to improve the reproducibility of the work that we publish. This form provides structure for consistency and transparency in reporting. For further information on Nature Portfolio policies, see our Editorial Policies and the Editorial Policy Checklist.

Statistics

For all statistical analyses, confirm that the following items are present in the figure legend, table legend, main text, or Methods section.

n/a	Confirmed	
☐	☒	The exact sample size (*n*) for each experimental group/condition, given as a discrete number and unit of measurement
☐	☒	A statement on whether measurements were taken from distinct samples or whether the same sample was measured repeatedly
☐	☒	The statistical test(s) used AND whether they are one- or two-sided<br>*Only common tests should be described solely by name; describe more complex techniques in the Methods section.*
☐	☒	A description of all covariates tested
☐	☒	A description of any assumptions or corrections, such as tests of normality and adjustment for multiple comparisons
☐	☒	A full description of the statistical parameters including central tendency (e.g. means) or other basic estimates (e.g. regression coefficient) AND variation (e.g. standard deviation) or associated estimates of uncertainty (e.g. confidence intervals)
☐	☒	For null hypothesis testing, the test statistic (e.g. F, t, r) with confidence intervals, effect sizes, degrees of freedom and P value noted<br>*Give P values as exact values whenever suitable.*
☒	☐	For Bayesian analysis, information on the choice of priors and Markov chain Monte Carlo settings
☒	☐	For hierarchical and complex designs, identification of the appropriate level for tests and full reporting of outcomes
☐	☒	Estimates of effect sizes (e.g. Cohen's *d*, Pearson's *r*), indicating how they were calculated

Our web collection on statistics for biologists contains articles on many of the points above.

Software and code

Policy information about availability of computer code

Data collection	No software was used for data collection
Data analysis	Whole exome sequencing data were analyzed using Samtools (0.1.19), BWA(0.7.15), Picard (1.141), GATK (3.8), Mutect (1.1.7), VarScan2 (2.4.3), Sequenza (2.1.2), PHYLIP (3.698), GISTIC2.0, PyClone-VI, Oncotator (1.9.9.0) and R package deconstructSigs (1.8.0).Whole genome sequencing data were analyzed by SvABA (1.1.3), TIDDIT (2.12.1), DELLY (0.8.7), AnnotSV(3.1), AmpliconArchitect (1.2_r2), Strelka2(2.9.10), and R packages deconstructSigs (1.8.0) and MutationalPatterns (3.2.0).RNA sequencing data were analyzed by HISAT2 (2.0.6), htseqcount (0.5.4), CIBERSORTx (https://cibersortx.stanford.edu/) and R packages edgeR (3.28.1), limma (3.42.2), clusterProfiler (3.14.3), fgsea (1.12.0), GSVA (1.34.0) and CellChat (1.1.3).TCGA skcm pan-cancer data were analyzed by GISTIC2.0, CIBERSORTx (https://cibersortx.stanford.edu/) , and R packages GSVA (1.34.0) and maftools (2.2.10). We conducted statistical analyses in R.4.02, Python 3.8.0, Python 2.7.17 and Prism.

For manuscripts utilizing custom algorithms or software that are central to the research but not yet described in published literature, software must be made available to editors and reviewers. We strongly encourage code deposition in a community repository (e.g. GitHub). See the Nature Portfolio guidelines for submitting code & software for further information.

March 2021

Data

Policy information about availability of data

All manuscripts must include a data availability statement. This statement should provide the following information, where applicable:

- Accession codes, unique identifiers, or web links for publicly available datasets
- A description of any restrictions on data availability
- For clinical datasets or third party data, please ensure that the statement adheres to our policy

The BAM files of WES, WGS, and RNA-seq data are deposited in the European Genome-phenome Archive (https://www.ebi.ac.uk/ega/) with the accession number EGAS00001006644.
TCGA skcm dataset was collected from cBioPortal (https://www.cbioportal.org/)

Human research participants

Policy information about studies involving human research participants and Sex and Gender in Research.

Reporting on sex and gender	Sex/gender was self-reported and not considered in the study design given that each cohort size was small.
Population characteristics	28 patients (age: 37~83) with BRAF mutant cutaneous melanoma treated by MAPKi and 7 patients (age: 42~80) with cutaneous melanoma treated by ICB
Recruitment	By physician and self referall
Ethics oversight	This study is approved by institutional review boards at UCLA and Vanderbilt-Ingram Cancer Center

Note that full information on the approval of the study protocol must also be provided in the manuscript.

Field-specific reporting

Please select the one below that is the best fit for your research. If you are not sure, read the appropriate sections before making your selection.

☒ Life sciences ☐ Behavioural & social sciences ☐ Ecological, evolutionary & environmental sciences

For a reference copy of the document with all sections, see nature.com/documents/nr-reporting-summary-flat.pdf

Life sciences study design

All studies must disclose on these points even when the disclosure is negative.

Sample size	The sample sizes in this study were determined by the number of melanoma metastases with high-quality DNA/RNA sample available.
Data exclusions	No data was excluded from the analysis
Replication	All attempts of replication were successful and indicated in the figure legends or methods
Randomization	Mice with similar tumor volumes were randomly selected into groups. Tumor measurements were not blinded.
Blinding	The warm autopsy melanoma cases and clinical cohorts were collected independently by clinical technicians not involved in the experimental design or data analysis.

Reporting for specific materials, systems and methods

We require information from authors about some types of materials, experimental systems and methods used in many studies. Here, indicate whether each material, system or method listed is relevant to your study. If you are not sure if a list item applies to your research, read the appropriate section before selecting a response.

Materials & experimental systems

n/a	Involved in the study
☐	☒ Antibodies
☒	☐ Eukaryotic cell lines
☒	☐ Palaeontology and archaeology
☐	☒ Animals and other organisms
☒	☐ Clinical data
☒	☐ Dual use research of concern

Methods

n/a	Involved in the study
☒	☐ ChIP-seq
☒	☐ Flow cytometry
☒	☐ MRI-based neuroimaging

Antibodies

Antibodies used

anti-GRIK4 (Invitrogen, MA5-31745); anti-GRIN1 (Abcam, ab109182); anti-GABRG1 (Invitrogen, PA5-99317); anti-GABRA2 (Invitrogen, PA5-106894); anti-IFNK (Novus, H00056832-M01); anti-IFNAR2 (Invitrogen, PA5-119915); Alexa Fluor Plus 488 (Invitrogen, A32723); Alexa Fluor 555 (Invitrogen, A-21429); anti-MRT-1 (Abcam, ab210546); anti-iNOS (Abcam, ab115819); anti-CD68 (Roche, 790-2931); anti-CD163 (Abcam, ab182422); anti-CD206 (Cell Signaling, 91992S); anti-SOX10 (Abcam, ab227680); OmniMap anti-Ms HRP (Roche, 760-4310); OmniMap anti-Rb HRP (Roche, 760-4311)

Validation

All antibodies are commercially available and are stated to be tested by the manufacturer for species reactivity to human. Information in Abcam, Invitrogen, Cell Signaling, Roche and Novus certificate of analyses. Abcam, Invitrogen, Cell Signaling, Roche and Novus antibodies are knock-out/knockdown validated or validated in cells with established levels of protein expression. The statements and validation data for each primary antibody for the species and application are also available on the manufacturer's website.
The specificity of anti-MRT-1 (Abcam, ab210546); anti-iNOS (Abcam, ab115819); anti-CD68 (Roche, 790-2931); anti-CD163 (Abcam, ab182422); anti-CD206 (Cell Signaling, 91992S); and anti-SOX10 (Abcam, ab227680) was further verified by using human melanoma tumor samples.

Animals and other research organisms

Policy information about studies involving animals; ARRIVE guidelines recommended for reporting animal research, and Sex and Gender in Research

Laboratory animals

Sex-matched NSG mice (4-6 weeks old male and female) were used for developing the patient-derived xenograft (PDX) models. Information on the gender of patients from which PDX models were derived is located in Supplementary Table 6.

Wild animals

The study did not involve wild animals.

Reporting on sex

self reported sex was not considered in the analysis due to limited sample sizes

Field-collected samples

The study did not involve samples collected from the field.

Ethics oversight

All mouse experiments were approval by the local institutional review boards and the UCLA Animal Research Committee

Note that full information on the approval of the study protocol must also be provided in the manuscript.
